# RareCapsNet: An explainable capsule network enables robust discovery of rare cell populations from large-scale single-cell transcriptomics

**DOI:** 10.1371/journal.pcbi.1013962

**Published:** 2026-08-10

**Authors:** Sumanta Ray, Snehalika Lall

**Affiliations:** 1 Data Science, The West Bengal National University of Juridical Sciences, Kolkata, West Bengal, India; 2 Human Genetics Unit, Indian Statistical Institute, Kolkata, West Bengal, India; Sun Yat-Sen University, CHINA

## Abstract

Large-scale single-cell RNA sequencing has created new opportunities for computational identification of rare cell populations from complex transcriptomic data. Improvements in sequencing throughput now enable the profiling of tens of thousands of cells, increasing the likelihood of capturing rare cell types. We develop RareCapsNet, a rare cell identification technique through capsule network in large single cell RNA-seq data. RareCapsNet aiming to leverage the landmark advantages of capsule networks in single cell domain, by identifying rare, poorly represented, or putative rare cell populations through markers genes explained from human-mind-friendly interpretation of lower-level (primary) capsules. We demonstrate the explainability of capsule network for identifying capsule-associated markers that are act as signature of certain cell population of rare type. A comprehensive evaluation in simulated and real life single cell data demonstrate the efficacy of RareCapsNet for finding out rare population in large scRNA-seq data. RareCapsNet outperforms the other state-of-the-art not only in specificity and selectivity for identifying rare cell types, it can also successfully extract transcriptomic signature of the cell population. We demonstrate RareCapsNet to the dataset of multiple batch, where the model can store the knowledge of one batch which can be transferred to find out rare cells of other batch without training the model. *Availability and Implementation:* RareCapsNet is available at: https://github.com/sumantaray/RareCapsNet.

## Author summary

Single-cell RNA sequencing allows researchers to study thousands of individual cells and understand how different cell types contribute to health and disease. However, some biologically important cells occur in very small numbers. These rare cells may include immune subtypes, cancer-related cells, progenitor cells, or specialized neuronal populations. Because they are poorly represented, standard computational methods may miss them or fail to explain which genes make them distinct. We developed RareCapsNet, a capsule-network-based method for detecting rare cell populations in large single-cell transcriptomic datasets. The model learns relationships between gene-expression patterns and cell types through capsule routing, which helps it identify rare or underrepresented cells more accurately. Importantly, RareCapsNet also provides an interpretable way to connect predicted rare cell types with marker genes by analyzing capsule-coupling patterns. We tested RareCapsNet on simulated and real single-cell datasets, including immune and brain datasets, and compared it with existing rare-cell detection methods. Our results show that RareCapsNet can improve rare-cell identification while also suggesting biologically meaningful genes associated with these rare populations.

## Introduction

Single-cell transcriptomics enables us to understand the cellular composition of complex tissues and organisms in single-cell resolution [1–3]. In-silico analysis of single cell data (downstream analysis) seeks considerable attention to the machine learning researchers in the last few years [3,4]. Recent technological advances and increases in throughput capabilities open up great new chances to discover rare cell types [5]. Rare cells (e.g., circulating endothelial cells, endothelial progenitor cells, circulating tumor cells, cancer stem cells, etc.) are minor cell types present in an organism, which play an important role in the pathogenesis of cancer, mediating immune responses, angiogenesis in cancer, and other diseases, etc. Algorithmic approaches for rare cell identification are scarce and not readily available within the standard downstream analysis pipeline [6]. This is mostly due to, (i) unavailability of reference datasets with known cellular composition, (ii) lack of gene selection algorithms having good discriminating capabilities between major and minor cell types, (iii) unavailability of supervised approaches that could accurately identify poorly covered cells (cells with little samples). For realizing and dealing with rare cell types in scRNA-seq, advanced and very sensitive machine learning approaches bear great promises.

To comprehensively characterize the major and minor cell types within a complex tissue, processing of several thousands of single cells are required [1]. In other words, larger sample size increases the probability of capturing minor subpopulation in a tissue. This is mostly due to the failure of the amplification stage of sequencing technology where large number of type-specific transcripts are remained undetected [7]. This results insufficient number of type-specific marker genes that often fail to influence downstream analysis. Recently, technological advances enabled the parallel profiling of tens of thousands of single cells, thanks to the droplet-based single-cell transcriptomics.

A key goal of single cell RNA-seq analysis is to annotate cells within the specific type as efficiently as possible. The most popular and typical standard pipeline of downstream analysis (such as Seurat V4 [8] and Scanpy [9]) does not guarantee the identification of rare cellular identities.

The standard and conventional process of downstream analysis failed to identify the minor subpopulation present in the data. The number of existing procedures dedicated to the rare cell identification is also scarce. Recently Wegmann et al. [10] highlighted a methodology gap for comprehensive analysis and identification of rare cellular identities from the scRNA-seq data. Few dedicated algorithms exist for identifying rare cellular identities. RaceID (rare cell-type identification) [11], GiniClust [12], FiRE (Finder of Rare Entities) [7], and CellSIUS (Cell Subtype Identification from Upregulated gene Sets) [10] are prominent among them. RaceID uses computationally expensive parametric modeling to detect outlier expression profiles. It utilized an unsupervised clustering method (k-means) to define cell clusters, which in turn are utilized to determine outlier events (cells). GiniClust utilized a straightforward two-step algorithm based on the identification of quality genes. It first selects informative genes using the Gini index and then performed a density-based clustering method (DBSCAN) to discover outlier cells. CellSIUS follows the overall theme of RaceId and Giniclust by performing a clustering based method to identify rare cells. It starts with an initial assignment of cells within different clusters and then updates the assignment by utilizing the expression of cluster-specific gene markers. Of note, all three methods utilized the clustering process to discriminate between major and minor cell types. The clustering is performed by computing the distance between each pair of cells, thereby suffers from huge computational cost resulting in slow and memory inefficient algorithms for oversized scRNA-seq data. FiRE produces a relatively fast and clustering-free algorithm for rare cell identification. It performs a sketching process based on hashing technique for projecting cells into low-dimensional bit signatures (hash code). This process gives a set of buckets where the rare cells share the same bucket with small number of other cells. It then computes a consensus rareness score based on several rareness estimates for each of the studied cells.

Despite recent progress, existing rare-cell detection methods remain largely clustering-dependent, scale poorly with increasing dataset size, and offer limited interpretability at the gene level. Moreover, their performance degrades substantially under extreme class imbalance and batch variability—conditions that are intrinsic to large-scale single-cell transcriptomic data. Here we introduced RareCapsNet, an intrepretable capsule network for identifying rare cells through cell specific gene markers. Notably, this approach largely and effectively masks all the aforementioned limitations associated with other rare cell detection approaches. Recently few studies highlight the potential explainability properties of the capsule network in several applications [6,13–15]. Here, we suggest to leverage the original trade marks of capsule networks for identifying transcriptomic signature for the cell population of rare type. RareCapsNet takes a stepwise approach which train, test and interpret the model in an organized and meaningful way. The motivation for using capsule networks is that it was able to successfully resolve analogous issues in image recognition, their original application. Thereby, they established landmark advantages in particular with respect to sustainable use of training data and human mind-friendly interpretation of results. This is possible through the interpretation of the primary capsule which are the fundamental building blocks of capsule networks. The meaning (interpretation) of primary capsules make us understand the signature features (genes) for cells of a particular type (also for rare type), which is the fundamental challenge for rare cell detection in large scRNA-seq data. We evaluated the efficacy of RareCapsNet on a number of real and simulated datasets and compare with several state-of-the-arts. RareCapsNet can accurately identify rare cell types from large scRNA-seq mouse brain dataset, dendritic subtypes of human blood dendritic cell sub-types using 68k single-cell expression profile.

## Results and discussions

### Workflow of RareCapsNet

**Fig 1:**
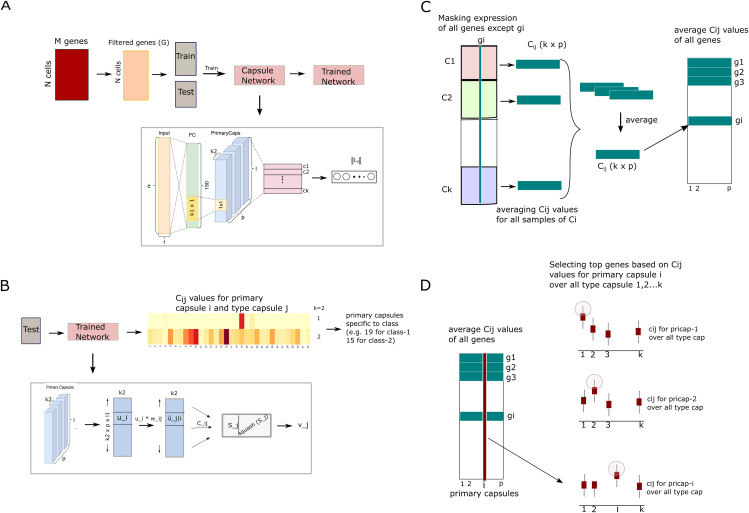
Overview of the RareCapsNet workflow. **(A)** Preprocessing of the scRNA-seq data. The input expression matrix contains *N* cells (rows) and *K* genes/features (columns). After filtering, normalization, and log-transformation, each individual cell is represented by a *K*-dimensional expression vector. **(B)** Capsule-network architecture. During training or inference, the input to the network is a *single-cell* expression vector. This vector is first passed through a fully connected layer, then through the primary capsule layer, and finally to the type capsule layer by dynamic routing. The number of type capsules equals the number of annotated cell types. **(C)** Interpretation of capsule associations using coupling coefficients between primary capsules and type capsules. Stronger coupling indicates that a given primary capsule contributes more strongly to the prediction of a particular cell type. **(D)** Gene-level attribution. For a selected primary capsule–type capsule pair, gene-specific coupling coefficients are computed by masked-input analysis to identify marker genes associated with the corresponding cell type.

Fig 1 describes the workflow of the whole analysis.

***A. Preprocessing the data*** See -A of Fig 1. Raw scRNA-seq data, obtained from public data sources are preprocessed and normalized using a transformation method (Linnorm) [16]. We keep cells having more than a thousand genes expression values (non zero values) and choose genes which having the minimum read count greater than 5 in at least 10% of the cells. *log*_2_ normalization is performed on the transformed matrix by adding one as a pseudo count.

***B. Model training and optimization***. Resulting data is divided into training, validation and test data sets. For exploration and adjustment of hyperparameters and optimizing the underlying capsule network architecture, we perform cross-validation runs. The resulting model consists of an ordinary fully connected layer, followed by a primary capsule and an output capsule layer (the connection between the latter two of which encompasses a dynamic routing procedure). Output capsules refer to the individual cell types that one can predict. The details architecture of the proposed model is shown in a separate box (panel-A, Fig 1). The input parameters, e.g., kernel size (k1) in first fully connected (FC) layer, kernel size (k2) of primary capsule layer, number of primary capsules (P) are fixed for all datasets, while number of cells (N), number of class/number of type capsules (k) depend on the datasets used here.

***C. Interpreting primary capsules.*** See panel-B of Fig 1. The key points of motivation of our method is the interpretability of essential building blocks of the underlying capsule model. We find out the primary capsules of a trained model that were in strong association with the corresponding class (here cell type). First we train the model with available training data and retain the model that gives best performance. Test samples are given as input to the trained network and coupling coefficients($c_{ij}$) between primary capsules and type (class) capsules are observed. Panel-B of Fig shows a heatmap demonstrating the association between two layers of capsules (here 32 primary in first layer and 2 type capsule in output layer) as an example. The output of this step is the index of activated primary capsules specific to the classes.

***D. Associating features (genes) with primary capsules***. See panel-C and -D of Fig 1. This step associates features (genes) with the activated primary capsules. We proposed an algorithm for identifying top genes that can activates a particular primary capsules. Given a primary capsule, which is known to be activated for a particular class (cell type), we identify features (genes) that are responsible for activation of the same primary capsule. The algorithm starts with masking all features except one and storing the average coupling coefficient by given the data as input to the trained network. The result of this step consist of a set of genes for a particular primary capsule that are known to be associated with a class capsule.

***E. Supervised setting and candidate novel-cell detection*** RareCapsNet is formulated primarily as a supervised rare-cell identification framework. During training, the model requires cell-type labels to learn associations between gene-expression patterns, primary capsules, and type capsules. Therefore, the method is most directly applicable to detecting rare or poorly represented cell types that are present in the training annotations or in a related reference dataset.

For cells from a new dataset, RareCapsNet assigns each cell to the type capsule with the largest activation. However, cells that do not match any known type capsule may show low maximum capsule activation, weak prediction confidence, or diffuse coupling across multiple type capsules. Such cells can be flagged as candidate outliers or putative novel populations for further analysis. We emphasize that these candidates should not be interpreted as definitive novel cell types without additional validation, such as clustering of the flagged cells, inspection of marker genes, comparison with public marker databases, and functional enrichment analysis. Thus, the current RareCapsNet framework supports supervised rare-cell detection and can highlight candidate outlier populations, while fully unsupervised or open-set novel cell-type discovery remains an important direction for future work.

The workflow in Fig 1 provides a conceptual overview of RareCapsNet; the detailed mathematical formulation of the architecture, dynamic routing procedure, and training objective is provided in the Method section under “Architecture and training objective of RareCapsNet.

### Explainability of capsules in rare cell detection

A simulation study is designed to show the explainibility of primary capsules for capturing the characteristics of rare cellular identities. Here we have shown one (or more) primary capsules are activated for a particular cell of rare type. For this, we have utilized splatter [17], a widely used tools for simulation of realistic scRNA-seq data.

#### Data generation.

Here we generate the data with four experimental setups:

S1: generated 1000 cells in two groups, with sample a ratio of 90:10, 95:5, 99:1 and 99.5:0.05 keeping low dropout rate (~0.2), proportions of differentially expressed (DE) genes as 40%, over 2000 genes

S2: generated two groups of cells, consisting 50% of the total (1000) cells in each group, over 2000 genes at a high dropout rate (~0.5), proportions of differentially expressed (DE) genes as 20%

S3: generated 1000 cells in three groups, with sample ratio of 80:10:10, 90:5:5, 98:1:1 and 99:0.05:0.05 keeping low dropout rate (~0.2), proportions of differentially expressed (DE) genes as 40%, over 2000 genes

S4: generated four equal-sized groups of 1000 cells over 2000 genes at a high dropout rate ~0.5, and proportion of DE genes as 20%.

S5: Thirteen cell types were generated to simulate a complex multi-class setting with multiple rare populations. Four cell types were defined as rare, with proportions 0.5%, 1%, 2%, and 5%, while the remaining nine cell types shared the remaining cells approximately equally. The target dropout rate was approximately 0.2, and 40% of genes were differentially expressed. This setting was repeated 10 times with different random seeds.

The details of the simulation settings are shown in Table 1.

**Table 1:** Synthetic datasets grouped by simulation settings (S1–S5). Each dataset varies by class ratio, dropout rate, and proportion of differentially expressed (DE) genes.

Group	Dataset	Sample Ratio	Dropout	Prop. of DE Genes	#Classes
S1	S1.1.	90:10	0.2	40	2
	S1.2.	95:5	0.2	40	2
	S1.3.	99:1	0.2	40	2
	S1.4.	99.5:0.05	0.2	40	2
S2	S2.1.	50:50	0.5	20	2
S3	S3.1.	80:10:10	0.2	40	3
	S3.2.	90:5:5	0.2	40	3
	S3.3.	98:1:1	0.2	40	3
	S3.4.	99:0.05:0.05	0.2	40	3
S4	S4.1.	25:25:25:25	0.5	20	4
S5	S5.1.	Samples of 4 classes < 10% of total cells, others are distributed equally	0.2	40	13

### Performance of RareCapsNet on simulated data

**Fig 2:**
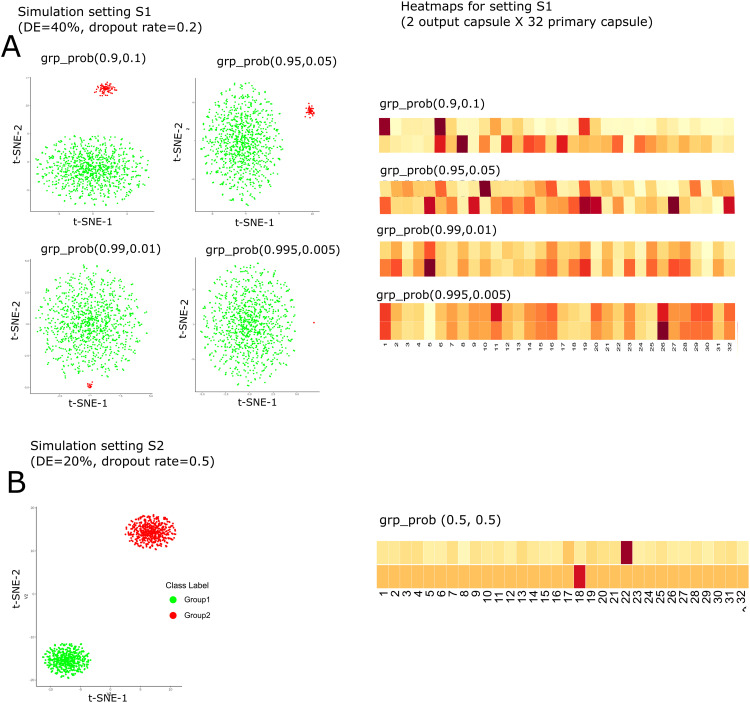
Coupling strength visualization under simulation settings S1 and S2. The panel A shows simulation setting S1, where two cell populations were generated under increasing class imbalance with sample ratios 90:10, 95:5, 99:1, and 99.5:0.5, using 40% differentially expressed genes and a dropout rate of 0.2. The left-side t-SNE plots show that the rare population becomes progressively smaller as the imbalance increases. The corresponding heatmaps on the right show coupling coefficients between 2 output capsules and 32 primary capsules for each sample-ratio setting. Darker red regions indicate stronger coupling, suggesting that specific primary capsules become strongly associated with the rare cell population. The panel B shows simulation setting S2, where two balanced cell populations were generated with sample ratio 50:50, 20% differentially expressed genes, and a higher dropout rate of 0.5. The t-SNE plot shows separation between the two simulated groups, while the heatmap illustrates the learned coupling pattern between the output capsules and primary capsules under high-dropout conditions. Overall, the Figure demonstrates that RareCapsNet learns class-specific capsule-routing patterns under both extreme imbalance and high-dropout simulation settings.

RareCapsNet can distinguish minor subpopulation of cells in all type of simulation settings (S1 to S5). simulated single cell data obtained from each setting is processed with RareCapsNet with a train-test split of 80:20. We store the coupling coefficient ($c_{ij}^{(}s)$) values for the test set applied on the fully trained model. To get a stable setup, we have applied the trained model multiple times (100 times here) on the test samples and obtained the average $c_{ij}$s. The main aim of this study is to find out the association of primary capsules with minor subpopulations of cells present in the data. For each simulation settings one (or more than one) primary capsules are noticed to be activated (see Fig 2) that explain the characteristics of cells of a particular type. For S1 and S2 setups the simulated data consist of two groups/classes, whereas for S3 and S4 the generated data has three groups/classes. S1 and S3 contain four simulated data with varying sample ratio, whereas for S2 and S4 setups sample ratio are fixed. The aim is to show the performance of RareCapsNet in diverse simulated data with fixed and varying number of samples. RareCapsNet produces one (or more than one) activated primary capsules for the minor subpopulations in each simulation settings (see Fig 2 for the results of simulation data S1 and S2). The activated primary capsules may be treated as marker of the generated cells of specific type. For example, in setting S1, the generated data with sample ratio 95:5, primary capsule 9, 19 and 26 are activated for the minor (5%) population, while primary capsule 10 is activated for the major (96%) population. For the other setups (e.g., S2, S3 and S4) the similar situations can be observed in the activation pattern of the primary capsules. The results for dataset S3, S4 and S5 are given in S1 Text. It may be happened that one activated primary capsule representing the samples of two or more types (e.g., The results for simulation settings are provided in Table D and Table E of S1 Text. In the S5 setting, primary capsule 32 showed in Table 3 high coupling with two cell types, indicating that one primary capsule may occasionally capture shared transcriptomic features across more than one rare population.).

We compared RareCapsNet with four established rare-cell detection methods: GiniClust, FiRE, RaceID, and CELCIUS, across five simulation settings S1–S5. The Tables D and E of S1 Text provide an extended simulation-based comparison of RareCapsNet with GiniClust, FiRE, RaceID, and CELCIUS using accuracy, sensitivity/recall, and ROC-AUC. RareCapsNet achieved the best overall performance in both two-class and multi-class rare-cell simulation settings. The gain in sensitivity/recall indicates that RareCapsNet is particularly effective in detecting minority cell populations under severe class imbalance. These results support the robustness of the proposed capsule-routing framework for rare-cell identification. As shown in, Table 2, RareCapsNet achieved strong rare-cell detection performance across the simulated datasets S1–S5. The simulation settings included both binary and multi-class rare-cell scenarios with varying degrees of class imbalance, dropout, and differential gene-expression proportions. RareCapsNet obtained the highest F1-score in most of the simulated settings, indicating its ability to recover minority cell populations under different rare-cell configurations. The improvement was particularly evident in highly imbalanced settings, such as S1.3, S1.4, S3.3, and S3.4, where rare populations were represented by very small numbers of cells. This suggests that the capsule-routing mechanism can preserve rare-cell-specific transcriptomic signals even when the minority class is poorly represented. Although RareCapsNet showed the best overall trend, some baseline methods performed comparably in selected settings. For example, RaceID showed slightly higher F1-score in S1.1, while FiRE performed marginally better in S3.1 and S3.2. Therefore, the results should not be interpreted as uniform superiority in every individual simulation condition, but rather as evidence that RareCapsNet provides robust and competitive rare-cell detection performance across a broad range of simulated rare-cell scenarios. Since F1-score alone may not fully describe model behavior under class imbalance, we further evaluated additional performance measures, including accuracy, sensitivity/recall, and ROC-AUC. These extended results are provided in Tables D and Table E of S1 Text. Table D of S1 Text reports the additional metrics for two-class simulated rare-cell settings, whereas Table E of S1 Text reports the corresponding metrics for multi-class and complex rare-cell simulation settings. Together, these supplementary analyses provide a more complete assessment of the classification performance and rare-cell sensitivity of RareCapsNet and the competing methods.

**Table 2:** F1-score performance of competing methods on synthetic datasets.

Dataset	F1 Score					#Classes
	RareCapsNet	GiniClust	FiRE	RaceID	CellSIUS	
S1.1.	0.89	0.76	0.80	**0.90**	0.88	2
S1.2.	**0.88**	0.78	0.81	0.84	0.80	2
S1.3.	**0.85**	0.68	0.80	0.82	0.74	2
S1.4.	**0.78**	0.59	0.68	0.72	0.69	2
S2.1.	**0.95**	0.92	0.94	0.91	0.88	2
S3.1.	0.91	0.90	**0.92**	0.89	0.87	3
S3.2.	0.89	0.86	**0.90**	0.87	0.86	3
S3.3.	**0.86**	0.74	0.85	0.79	0.78	3
S3.4.	**0.79**	0.70	0.68	0.61	0.59	3
S4.1.	0.90	0.89	0.90	**0.91**	0.91	4
S5.1.	**0.93**	0.87	0.82	0.80	0.89	13

### Ablation analysis of capsule-network parameters

To assess the sensitivity of RareCapsNet to key architectural choices, we performed an ablation study by varying the number of primary capsules and the number of routing iterations. The experiments were conducted using the S1.2 simulation setting, which contains two cell populations with a 95:5 class ratio and therefore provides a controlled but challenging setting for rare-cell identification.

As shown in Table 3, increasing the number of primary capsules from 8 to 32 improves rare-cell detection performance, suggesting that additional capsules increase the representational capacity needed to capture minority-cell-specific expression patterns. However, increasing the number of primary capsules to 64 does not provide further improvement and slightly reduces the F1-score, possibly due to overparameterization or reduced capsule specialization. Similarly, increasing the number of routing iterations improves performance up to 3 iterations, after which the performance saturates. These results justify the use of 32 primary capsules and 3 routing iterations in all subsequent experiments. Additional details and plots for the ablation analysis are provided in the S1 Text.

**Table 3:** Ablation analysis of RareCapsNet hyperparameters. F1-scores are reported for rare-cell detection under the S1.2 simulation setting. The results support the use of 32 primary capsules and 3 routing iterations in the main experiments.

Ablation setting	Parameter value	F1-score
Number of primary capsules	8	0.79
	16	0.84
	32	**0.91**
	64	0.89
Number of routing iterations	1	0.82
	2	0.88
	3	**0.91**
	4	0.90

### Robust identification of rare cell type in poorly covered cells

To evaluate the performance of RareCapsNet in scenarios where rare cell populations are poorly covered or have low sample representation, we tested our model on two scRNA-seq datasets: *Jurkat* (a T-cell leukemia cell line) and *CBMC* (Cord Blood Mononuclear Cells).

### Jurkat–293T mixed-cell experiment

The Jurkat cell line, originally established from the peripheral blood of a 14-year-old boy with T-cell leukemia, is an immortalized human T lymphocyte line widely used to study T-cell signaling and leukemia [7]. The Jurkat–293T mixture contains two human cell lines, Jurkat and HEK 293T, with approximately 3,200 cells and approximately 2,000 genes after preprocessing. We also clarify that Jurkat cells constitute approximately 2.5 of the total population and were treated as the rare target population, while 293T cells formed the majority class. In our experiments, we utilized a mixed dataset comprising Jurkat and 293T cells, where Jurkat cells constituted approximately 2.5% of the total population. This artificial dilution simulates conditions with extremely low representation of the minority class.

**Fig 3:**
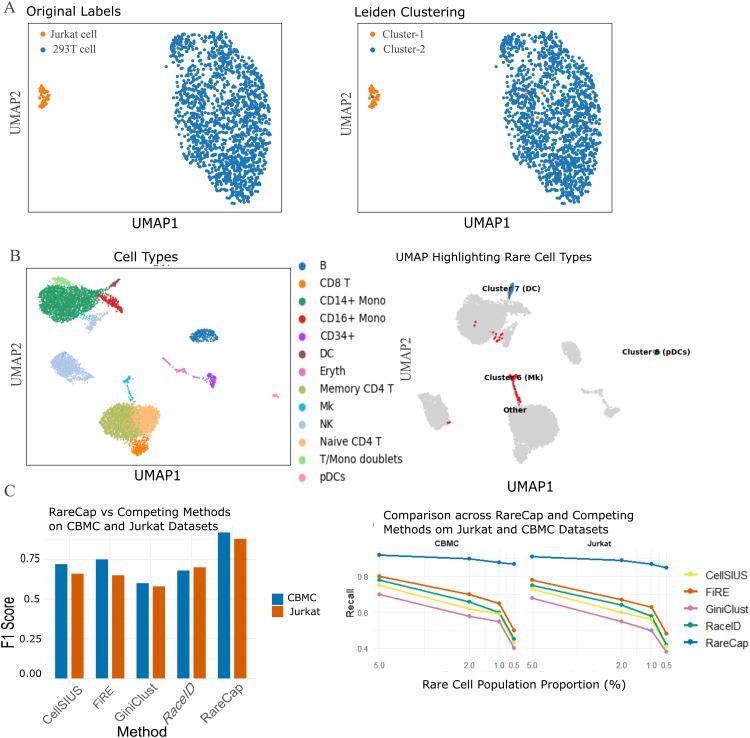
Robust identification of poorly covered rare cell populations. **(A)** UMAP visualization of the Jurkat–293T mixed dataset showing the original cell labels (left) and Leiden clustering results (right). While Leiden clustering fails to distinctly separate the rare Jurkat population, RareCapsNet successfully captures the minority class through capsule-based representations. **(B)** UMAP visualization of the CBMC dataset highlighting rare immune cell types, including megakaryocytes (Mk), dendritic cells (DCs), and plasmacytoid dendritic cells (pDCs). RareCapsNet identifies these rare populations through distinct primary capsule activations, even when their abundance is below 1%. **(C)** Quantitative comparison of RareCapsNet against state-of-the-art rare cell detection methods (FiRE, GiniClust, RaceID, and CellSIUS) on the Jurkat and CBMC datasets. Left: F1-scores across methods. Right: recall as a function of decreasing rare cell population proportion. RareCapsNet consistently achieves superior performance, maintaining high recall and precision even at extreme rarity levels (down to 0.5%).

RareCapsNet was trained on 80% of the cells, with the remaining 20% reserved for testing (with a stratified setting). The model consistently identified key activated primary capsules linked to the Jurkat cells and successfully highlighted interpretable gene markers. Compared to FiRE, CellSIUS, GiniClust, and RaceID, RareCapsNet exhibited superior F1-score and precision in identifying the rare Jurkat cell subtype (see Fig 3, panel-C). Notably, RareCapsNet maintained a high recall even at a 0.5% population ratio, demonstrating robustness in extremely low prevalence settings. It can also be noted from Fig 3A that the Leiden clustering result largely separates the two transcriptomic groups, although a few cells show locally ambiguous assignments. Such ambiguity can arise because Leiden clustering is performed on a nearest-neighbor graph, whereas UMAP provides a two-dimensional projection of that graph; therefore, apparent visual proximity in UMAP may not always exactly match the graph connectivity used for clustering. In addition, rare cells represented by very few samples may be more sensitive to local neighborhood uncertainty and expression noise. This observation further motivates the use of RareCapsNet, which combines supervised capsule representations with coupling-based signals for improved rare-cell detection beyond visual clustering alone.

### CBMC rare immune-cell experiment

Cord Blood Mononuclear Cells (CBMCs) are derived from umbilical cord blood and encompass a diverse array of immune cell types, including T cells, B cells, natural killer (NK) cells, monocytes, dendritic cells (DCs), megakaryocytes (Mk), and hematopoietic stem and progenitor cells (HSPCs). In our dataset, the most abundant cell types included CD14^+^” monocytes (2293 cells), memory CD4 + T cells (1791), and naïve CD4^+^ T cells (1248). In contrast, several clinically relevant cell types were present in very low numbers: Mk (96 cells), DCs (70), and plasmacytoid dendritic cells (pDCs, 49). These rare populations accounted for less than 1% of the total cell count, making their identification especially challenging.

To evaluate RareCapsNet under such conditions of extreme data imbalance, we trained the model on 80% of the CBMC data and retained 20% for testing. RareCapsNet demonstrated a remarkable ability to detect these rare cell types by learning interpretable and biologically grounded representations. Specifically, it successfully activated distinct primary capsules corresponding to Mk (cluster 6), DCs (cluster 7), and pDCs (cluster 8), capturing lineage-specific gene expression patterns.

In comparison with state-of-the-art rare cell detection tools such as FiRE, CellSIUS, GiniClust, and RaceID, RareCapsNet achieved superior F1-scores and precision, particularly on the rare subsets (see Fig 3, panel-C). Notably, the model maintained high recall even at population ratios as low as 0.5%, underscoring its robustness and sensitivity in detecting biologically meaningful rare populations (see Fig 3, panel-C). These results affirm RareCapsNet’s utility in uncovering rare yet critical immune cell subsets in complex and heterogeneous single-cell datasets.

### Identification of dendritic cells in PBMC68k

We next applied RareCapsNet to the PBMC68k dataset from 10x Genomics, which includes over 68,000 peripheral blood mononuclear cells with annotated immune cell types. A known challenge in this dataset is the identification of dendritic cell (DC) subtypes, which are typically present in very low abundance and are poorly separated using conventional clustering tools.

RareCapsNet was trained using a subset of the data and tested on the remaining cells. Through analysis of the coupling coefficients between primary and type capsules, the model identified distinct primary capsules strongly associated with DCs and their subtypes (e.g., pDCs and conventional DCs). Marker gene identification via coupling-based relevance scoring revealed known DC markers such as CLEC4C and LILRA4, as well as putative novel markers supported by literature.

RareCapsNet’s performance in dendritic cell identification outperformed GiniClust and CellSIUS, especially in terms of precision, while offering the added benefit of interpretable marker gene discovery.

### Results on Mouse brain data set [18]

We further evaluated RareCapsNet using the Zeisel mouse brain dataset [18], a complex scRNA-seq dataset comprising over 3,000 single cells from mouse cortex and hippocampus. The dataset contains well-annotated neuronal and non-neuronal cell types, including rare cell subtypes such as neurogliaform cells and ependymal cells.

RareCapsNet identified rare subpopulations through distinct primary capsule activations. These capsule activations corresponded to biologically meaningful gene sets associated with rare cell identities, including Reln, Ptn, and Ndnf—genes known to be enriched in neurogliaform cells.

Our model achieved an F1-score of 0.88 for rare neuronal subtypes, compared to 0.73 (RaceID), 0.69 (CellSIUS), and 0.62 (GiniClust), underscoring RareCapsNet’s ability to resolve fine-grained heterogeneity in complex neural tissues. Notably, the coupling-based interpretation also highlighted gene markers that support the functional differentiation of interneuron subtypes.

### Rare cell identification in different batches of data

To examine the generalizability of RareCapsNet across independently generated single-cell datasets, we evaluated the model in a cross-dataset transfer setting using Yan [19], Pollen [20], Darmanis [21], and CBMC [22]. These datasets differ in tissue origin, sequencing protocol, number of annotated cell types, and overall expression distribution, thereby providing a challenging setting for evaluating whether learned capsule activations retain biological relevance across datasets.

Before transfer, each dataset was processed using a consistent preprocessing pipeline, including library-size normalization, log-transformation, and selection of highly variable genes. Because different datasets may contain different gene sets, we did not directly transfer the model using unmatched input features. Instead, for each source–target transfer experiment, the input feature space was restricted to the common intersecting genes shared by the source and target datasets. The intersected gene expression matrices were ordered identically before being provided to the trained RareCapsNet model. Thus, all transfer experiments were performed in a matched gene space.

We adopted a transfer setting in which RareCapsNet was trained on a source dataset and then applied to a target dataset without additional retraining or fine-tuning. No explicit feature-alignment layer, domain-adaptation module, or transfer mapping layer was used. It is important to note that RareCapsNet is supervised in its current formulation. The number of type capsules is fixed by the number of annotated cell types in the source training dataset. Therefore, direct transfer is meaningful only for cell types that are shared or harmonized between the source and target datasets. If the target dataset contains a cell type that was not present during training, the current model cannot assign it to a new type capsule. Such cells may instead show low maximum type-capsule activation, weak prediction confidence, or diffuse coupling across multiple type capsules, and can be treated only as candidate outliers or putative novel populations requiring further validation. Thus, the present cross-dataset analysis evaluates transferability of learned capsule representations under a shared-gene and label-harmonized setting, rather than fully open-set discovery of unseen cell types. The goal of this analysis was therefore to assess whether capsule activations learned from one dataset remain informative when evaluated on a related dataset represented using the shared gene space.

As summarized in Table 4, RareCapsNet retained strong rare-cell detection performance in the evaluated cross-dataset settings. The model achieved greater than 85% accuracy when transferring from Darmanis to Pollen and 91% accuracy when transferring from CBMC to Yan. The learned primary capsule activations also remained qualitatively consistent across source and target datasets, suggesting that capsule routing can capture transferable expression patterns associated with rare or underrepresented cellular identities.

**Table 4:** Cross-dataset transfer performance of RareCapsNet. For each transfer experiment, RareCapsNet was trained on the source dataset and applied directly to the target dataset using the intersecting shared gene set. No retraining, fine-tuning, or additional feature-alignment layer was used.

Source dataset	Target dataset	Feature-space handling	Retraining / fine-tuning	Transfer performance
Darmanis	Pollen	Intersecting shared genes	No	>85% accuracy
CBMC	Yan	Intersecting shared genes	No	91% accuracy

However, we emphasize that the current transfer experiment assumes the availability of a sufficiently large shared gene set between datasets. The present implementation does not include a dedicated gene-space alignment module for datasets with largely non-overlapping gene panels. Therefore, cross-platform transfer across substantially different feature spaces remains a limitation of the current framework. Future extensions may incorporate domain-adaptation layers, gene-embedding-based feature alignment, or batch-aware capsule routing to improve transferability across heterogeneous single-cell resources.

### Comparison with deep learning-based single-cell methods

To further benchmark RareCapsNet against modern deep learning-based single-cell analysis frameworks, we compared its rare-cell detection performance with scVI [23], scANVI [24], CellAssign [25], and ItClust [26], in addition to the classical rare-cell detection methods considered above. scVI is a variational autoencoder-based generative model for single-cell transcriptomics, while scANVI extends this framework for semi-supervised annotation and dataset harmonization. CellAssign uses a probabilistic marker-informed framework for assigning cells to cell types, whereas ItClust uses neural network-based transfer learning for clustering and cell-type classification.

These methods were evaluated on representative real datasets, including PBMC68k, CBMC, Zeisel mouse brain, and a cross-batch transfer setting. For deep learning-based methods, the learned latent representations or predicted cell-type labels were used for rare-cell classification, and performance was assessed using F1-score for the rare cell population.

As shown in Table 5, deep learning-based methods generally improve over classical rare-cell detection approaches, particularly in the PBMC68k and cross-batch settings. However, RareCapsNet consistently achieves the highest F1-score across all datasets. The improvement is especially notable in the cross-batch transfer setting, where RareCapsNet maintains an F1-score of 0.78, compared with 0.72 for scANVI and 0.75 for ItClust. This suggests that capsule-based routing preserves rare-cell-specific representations more effectively under batch variability.

**Table 5:** Comparison of RareCapsNet with classical and deep learning-based single-cell methods. F1-scores are reported for rare-cell detection across representative real datasets and a cross-batch transfer setting. Classical rare-cell tools include RaceID, GiniClust, and FiRE, while deep learning-based baselines include scVI, scANVI, CellAssign, and ItClust. RareCapsNet achieves the highest F1-score across all settings, indicating improved sensitivity for rare cell populations under both within-dataset and cross-dataset evaluation.

Dataset / Setting	RareCapsNet	RaceID	GiniClust	FiRE	scVI	scANVI	CellAssign	ItClust
PBMC68k	**0.80**	0.72	0.67	0.78	0.76	0.78	0.74	0.79
CBMC	**0.81**	0.68	0.62	0.74	0.74	0.75	0.72	0.77
Zeisel brain	**0.84**	0.69	0.65	0.76	0.70	0.71	0.68	0.74
Cross-batch transfer	**0.78**	0.62	0.60	0.73	0.69	0.72	0.70	0.75

The superior performance of RareCapsNet may be attributed to two complementary properties. First, the dynamic routing mechanism enables primary capsules to specialize toward rare cell-associated expression patterns rather than being dominated by abundant cell types. Second, the coupling-based attribution mechanism provides an explicit link between rare-cell prediction and gene-level signals. In contrast, general-purpose latent representation methods such as scVI and scANVI learn useful low-dimensional embeddings but are not explicitly optimized to route rare-cell-specific information into interpretable type capsules. Similarly, CellAssign and ItClust provide useful annotation frameworks, but their performance may depend on marker availability, reference quality, or dataset-specific tuning. These results place RareCapsNet within the broader landscape of modern deep learning methods for single-cell analysis and demonstrate its advantage for interpretable rare-cell identification.

### Computational cost and scalability

Because capsule networks involve dynamic routing, we evaluated the computational cost of RareCapsNet relative to commonly used deep learning-based single-cell methods. On a PBMC68k-sized dataset, RareCapsNet required approximately 25 minutes of training time and 8.5 GB peak GPU memory on an NVIDIA RTX 3090 GPU with 24 GB memory. This runtime and memory usage were moderately higher than those of scVI, scANVI, CellAssign, and ItClust in our benchmark, reflecting the additional computational overhead introduced by iterative routing between primary capsules and type capsules.

The main computational cost of RareCapsNet arises from the routing operation and, separately, from the post hoc gene-attribution step. During model training, the number of primary capsules and routing iterations were fixed across experiments, which kept the training procedure practically scalable for datasets in the 50k–100k cell range after highly variable gene selection. Under this fixed architecture, runtime increased approximately linearly with the number of cells and input genes. The gene-attribution step is more computationally intensive because it requires repeated forward passes using masked gene inputs, however, this analysis is performed after training and can be parallelized across genes.

A detailed runtime and memory comparison is provided in S1 Text (section “Runtime, memory usage, and scalability”) and Table-B of S1 Text. Future optimization may include sparse routing, shared transformation matrices, mini-batch-level routing approximation, and GPU-parallelized gene-attribution computation to support atlas-scale single-cell datasets.

### Marker gene analysis

To further support the biological interpretability of the capsule-associated genes, we performed marker overlap and functional enrichment analyses. The top-ranked genes identified by RareCapsNet were compared with public marker resources, including CellMarker, PanglaoDB, and curated PBMC or brain cell-type annotations [27,28]. In addition, capsule-associated gene lists were analyzed using GO Biological Process and KEGG pathway annotations [29,30].

The recovered capsule-associated genes showed clear agreement with known rare-cell markers and biologically meaningful functions. For example, pDC-associated capsules recovered *CLEC4C*, *LILRA4*, *TCF4*, and *IRF7*, which are associated with plasmacytoid dendritic cell identity and interferon response. Dendritic-cell-associated capsules recovered *FCER1A*, *CST3*, and *HLA-DRA*, while megakaryocyte-associated capsules recovered *PF4*, *PPBP*, *ITGA2B*, and *GP9*. Functional enrichment analysis further showed that these capsule-associated genes were enriched for cell-type-relevant biological processes, including type I interferon signaling for pDCs, antigen processing and presentation for dendritic cells, and platelet activation for megakaryocytes.

As summarized in Table 6, RareCapsNet-derived capsule-associated genes are not merely predictive features but show substantial agreement with known marker resources and enriched biological processes. For top-ranked genes that were not included in canonical marker lists, we now refer to them as putative capsule-associated genes. This more conservative interpretation avoids overclaiming novelty while retaining the biological value of the capsule-based gene attribution framework. Additional details on the marker-resource overlap analysis, GO/KEGG enrichment procedure are provided in the section “Extended biological validation of capsule-associated marker genes.” of S1 Text.

**Table 6:** Biological validation of capsule-associated marker genes. Representative top-ranked genes identified by RareCapsNet are shown together with marker-resource overlap and enriched biological functions. Genes not included in canonical annotation lists are interpreted as putative capsule-associated genes rather than definitive novel biomarkers.

Rare cell population	Top capsule-associated genes	Marker-resource support	Representative enriched functions
pDCs	*CLEC4C*, *LILRA4*, *TCF4*, *IRF7*	CellMarker / PanglaoDB / PBMC markers	Type I interferon signaling; antiviral response; innate immunity
Dendritic cells	*FCER1A*, *CST3*, *HLA-DRA*, *LST1*	CellMarker / PanglaoDB / PBMC markers	Antigen processing and presentation; leukocyte activation
Megakaryocytes	*PF4*, *PPBP*, *ITGA2B*, *GP9*	CellMarker / PanglaoDB / blood-cell markers	Platelet activation; platelet degranulation; coagulation
Neurogliaform/interneuron-like cells	*RELN*, *NDNF*, *GAD1*, *GAD2*	Curated brain cell-type annotations	GABAergic synaptic signaling; neuronal differentiation

## Method

In this section, we first present the theoretical foundations underlying the proposed RareCapsNet framework, providing a formal description of the capsule-based modeling of gene–cell type relationships and a rigorous justification of the gene attribution strategy. We introduce precise definitions and state key propositions and theorems (with proof sketches) that characterize the behavior of coupling coefficients, their stability under class imbalance, and their role in identifying rare cell populations.

Subsequently, we describe the algorithmic procedures for computing gene-specific coupling coefficients and for statistically extracting marker genes associated with rare capsules.

### Architecture and training objective of RareCapsNet

We provide here the formal architecture and training objective of RareCapsNet. Let $X\in {\mathbb{R}}^{N\times K}$ denote the preprocessed single-cell expression matrix, where *N* is the number of cells and *K* is the number of genes or selected highly variable genes. For a single cell *s*, the input vector is


$$\begin{array}{c} x_{s}\in {\mathbb{R}}^{K}. \end{array}$$ (1)

Let *T* denote the number of annotated biological cell types. RareCapsNet contains *T* output capsules, referred to as type capsules, where each type capsule corresponds to one predicted cell type. The number of primary capsules is fixed at *J* = 32 in the final architecture, based on the ablation analysis reported in Table 3.

The input vector is first projected through a fully connected layer:


$$\begin{array}{c} h_{s}=\phi (W_{f}x_{s}+b_{f}), \end{array}$$ (2)

where $W_{f}\in {\mathbb{R}}^{H\times K}$ and $b_{f}\in {\mathbb{R}}^{H}$ are trainable parameters, *H* is the hidden dimension, and ϕ(·) denotes a nonlinear activation function.

The primary capsule layer consists of *J* capsules. The output of the *j*-th primary capsule is


$$\begin{array}{c} u_{j}(s)=squash(W_{j}^{\left(p\right)}h_{s}+b_{j}^{\left(p\right)}),\;j=1,...,J, \end{array}$$ (3)

where $u_{j}(s)\in {\mathbb{R}}^{d_{p}}$ is the vector output of primary capsule *j*. The squash function is defined as


$$\begin{array}{c} squash(z)=\frac{\|z{\|}^{2}}{1+\|z{\|}^{2}}\frac{z}{\left\|z\right\|}. \end{array}$$ (4)

This nonlinearity constrains capsule vector lengths to lie between 0 and 1, so that longer vectors indicate stronger capsule activation.

For each primary capsule *j* and type capsule *i*, RareCapsNet computes a prediction vector


$$\begin{array}{c} {\hat{u}}_{i|j}(s)=W_{ij}^{\left(t\right)}u_{j}(s),\;i=1,...,T,\;j=1,...,J, \end{array}$$ (5)

where $W_{ij}^{\left(t\right)}$ is a trainable transformation matrix.

Dynamic routing is used to determine the coupling strength between primary capsules and type capsules. Routing logits are initialized as $b_{ij}=0$. At routing iteration *r*, coupling coefficients are computed using a softmax over type capsules:


$$\begin{array}{c} c_{ij}^{\left(r\right)}=\frac{\exp(b_{ij}^{\left(r\right)})}{\sum_{i^{\prime }=1}^{T}\exp(b_{i^{\prime }j}^{\left(r\right)})}. \end{array}$$ (6)

For each type capsule *i*, the input vector is computed as


$$\begin{array}{c} s_{i}^{\left(r\right)}=\sum_{j=1}^{J}c_{ij}^{\left(r\right)}{\hat{u}}_{i|j}(s), \end{array}$$ (7)

and the output of the type capsule is


$$\begin{array}{c} v_{i}^{\left(r\right)}=squash(s_{i}^{\left(r\right)}). \end{array}$$ (8)

The routing logits are updated using agreement between the prediction vector and the type capsule output:


$$\begin{array}{c} b_{ij}^{\left(r+1\right)}=b_{ij}^{\left(r\right)}+{\hat{u}}_{i|j}(s{)}^{⊤}v_{i}^{\left(r\right)}. \end{array}$$ (9)

In all experiments, we used three routing iterations, as supported by the ablation analysis.

After routing, the predicted cell type for cell *s* is obtained from the type capsule with the largest vector norm:


$$\begin{array}{c} {\hat{y}}_{s}=\text{arg}\max_{i\in \{1,...,T\}}\|v_{i}\|. \end{array}$$ (10)

RareCapsNet is trained using the standard capsule margin loss. Let $T_{i}(s)=1$ if the true cell type of sample *s* is *i*, and $T_{i}(s)=0$ otherwise. The loss for sample *s* is


$$\begin{array}{c} ℒ(s)=\sum_{i=1}^{T}\left[T_{i}(s)\max(0,m^{+}-\|v_{i}\|{)}^{2}+\lambda (1-T_{i}(s))\max(0,\|v_{i}\|-m^{-}{)}^{2}\right], \end{array}$$ (11)

where *m*^+^ and *m*^-^ are positive and negative margins, respectively, and λ controls the contribution of absent cell types.

The model was trained using mini-batch gradient-based optimization. For each dataset, cells were divided into training, validation, and test sets. The training set was used to optimize the network parameters, the validation set was used for model selection and hyperparameter checking, and the test set was used only for final evaluation. After training, the coupling coefficients between primary capsules and type capsules were extracted for interpretation and for computing gene-specific coupling coefficients, as described in the following subsections.

### Definition of primary capsules and type capsules

In RareCapsNet, a *primary capsule* refers to an intermediate trainable vector-valued unit that learns latent gene-expression patterns from the input single-cell expression profile. Primary capsules should not be interpreted as pre-defined feature groups, gene modules, or biological cell types. Instead, they are learned representations whose specialization emerges during training through the dynamic routing procedure. The number of primary capsules is therefore a model hyperparameter; in the final architecture, we use *J* = 32 primary capsules based on the ablation analysis.

A *type capsule*, in contrast, refers to an output capsule corresponding to a predicted biological cell type. If a dataset contains *T* annotated cell types, the model contains *T* type capsules. The length of each type capsule output vector represents the activation strength for the corresponding cell type, and the predicted label is assigned to the type capsule with the largest norm. The coupling coefficient $c_{ij}$ measures the routing strength from primary capsule *j* to type capsule *i*, and is later used for model interpretation and gene-attribution analysis.

### A note on the dynamic routing procedure

Dynamic routing in RareCapsNet is an iterative agreement-based mechanism that determines how strongly each primary capsule contributes to each type capsule. Equation Equation (6) defines only one component of this procedure: the softmax normalization of routing logits into coupling coefficients. The complete routing procedure consists of four steps. First, each primary capsule produces a prediction vector ${\hat{u}}_{i|j}$ for every type capsule. Second, the routing logits $b_{ij}$ are converted into coupling coefficients $c_{ij}$ using the softmax function. Third, each type capsule receives a weighted sum of prediction vectors and applies the squash nonlinearity to produce its output vector. Fourth, the routing logits are updated according to the agreement between the prediction vector and the type capsule output. This procedure is repeated for a fixed number of routing iterations. In our final model, we use three routing iterations, as supported by the ablation analysis.

### Supervised setting and candidate outlier detection

RareCapsNet is primarily formulated as a supervised rare-cell identification framework. During training, the model requires cell-type labels to learn the association between gene-expression patterns, primary capsules, and type capsules. Therefore, the current implementation is most directly applicable to rare, poorly covered, or underrepresented cell types that are present in the training annotation or in a related reference dataset.

For a new dataset, RareCapsNet assigns each cell to the type capsule with the largest activation. If a cell does not match any learned type capsule, it may show low maximum type-capsule activation, weak prediction confidence, or diffuse coupling across multiple type capsules. Such cells may be flagged as candidate outliers or putative novel populations for further downstream analysis. However, these candidates should not be interpreted as definitive novel cell types without additional validation, such as clustering of flagged cells, marker-gene inspection, comparison with public marker databases, and functional enrichment analysis. Thus, the present RareCapsNet framework supports supervised rare-cell detection and candidate outlier flagging, while fully unsupervised or open-set novel cell-type discovery remains a future extension.

### Theoretical foundations of RareCapsNet gene attribution

First we formalize our gene attribution method and provide theoretical justification here. We introduce key definitions and propositions (with proofs) that supports the interpretation of coupling coefficients and the identification of marker genes.

**Definition 1** (Gene-Specific Coupling Coefficient). Let *S* be the set of training single-cell samples, with each sample s∈S represented by a *K-*dimensional expression vector (s[1],...,s[K]) across *K* genes. For a given trained RareCapsNet network, denote by $c_{ij}^{,s}$ the coupling coefficient between primary capsule j and type capsule i when the network processes sample *s* (through the dynamic routing procedure). For any gene $g_{k}$ (with k∈1,...,K), we construct a masked input $s^{k}$ from *s* by retaining only the expression of $g_{k}$ and setting all other gene expression values to 0. Let $c_{ij}^{,k,s}$ be the resulting coupling coefficient between primary capsule j and type capsule *i* when the network is fed $s^{k}$. We then define the gene-specific coupling coefficient for gene $g_{k}$ as the average coupling (with equal weight across cell types) over all training samples:


$$\begin{array}{c} c_{ij}^{k}=\frac{1}{T}\sum_{t=1}^{T}\frac{1}{N(t)}\sum_{\begin{array}{c} s\in S\\ t(s)=t \end{array}}c_{ij}^{k,s} \end{array}$$ (12)

where *T* denotes the number of cell types (and hence the number of type capsules, i=1,...,T)*, t(s)* denotes the cell type label of sample *s.* Here*, N(t)* denotes the number of cells belonging to cell type *t.* The inner average $\frac{1}{N(t)}\sum_{\begin{array}{c} s\in S\\ t(s)=t \end{array}}c_{ij}^{k,s}$ computes the mean gene-specific coupling within each cell type, while the outer average $\frac{1}{T}\sum_{t=1}^{T}$ gives equal contribution to each cell type. This stratified averaging prevents abundant cell types from dominating the attribution score and allows rare cell types to contribute equally to the final gene-specific coupling coefficient.

### Coupling-based definition of explainability

In RareCapsNet, explainability is defined through dynamic-routing coupling coefficients rather than through ordinary correlation. The coupling coefficient $c_{ij}$ measures the fraction of information from primary capsule *j* that is routed to type capsule *i* during prediction. A large value of $c_{ij}$ therefore indicates that primary capsule *j* contributes strongly to the prediction of cell type *i*. To obtain gene-level explanations, we compute gene-specific coupling coefficients by masking all genes except a single gene $g_{k}$ and measuring the resulting coupling between primary capsule *j* and type capsule *i*. Genes with unusually large gene-specific coupling values are interpreted as capsule-associated genes for the corresponding cell type.

The factor 1/*N*(*t*) in Eq. (12) averages the coupling coefficients over samples within cell type *t*. This prevents cell types with larger sample sizes from dominating the gene-specific coupling score. The outer factor 1/*T* then assigns equal weight to each cell type. Therefore, Eq. (12) implements a stratified averaging scheme that is especially important in rare-cell detection, where cell-type frequencies are highly imbalanced.

This definition formalizes the intuition that $c_{ij}^{k}$ measures how strongly gene $g_{k}$ contributes to activating the link from primary capsule *j* to type capsule *i*, aggregated over the entire training set with a stratified (per-cell-type) averaging. Next, we establish some key properties of the coupling coefficients arising from the dynamic routing algorithm in the RareCapsNet network.

**Proposition 1** (Normalization and Interpretability of Couplings). *For any input sample s (or masked sample* $s^{k}$*) passed through the MarkerCapsule network, the dynamic routing algorithm produces coupling coefficients* $c_{ij}^{,s}$ *satisfying:*

*Normalization:* $\sum_{i=1}^{T}c_{ij}^{,s}=1$ *for each primary capsule* j=1,...,J*. In other words, for a fixed primary capsule j, its coupling coefficients to all T type capsules form a probability distribution.*

*Preference for Alignment: If the output (“prediction vector”) of primary capsule j aligns well with type capsule i for the given input s, then* $c_{ij}^{,s}$ *will be correspondingly large. Conversely, if there is little or no agreement between capsule j’s features and the characteristics of type i,* $c_{ij}^{,s}$ *remains small. Thus,* $c_{ij}^{,s}$ *can be interpreted as the fraction of capsule j’s output that is assigned to explaining the presence of cell type i in sample s.*

*Proof.* We recall the standard dynamic routing procedure used in capsule networks. Fix an input sample *s* (or masked sample $s^{k}$). Let ${\hat{𝐮}}_{i|j}(s)\in {\mathbb{R}}^{d}$ denote the *prediction vector* produced by primary capsule *j* for type capsule *i*, i.e.,


$$\begin{array}{c} {\hat{𝐮}}_{i|j}(s)={𝐖}_{ij}{𝐮}_{j}(s), \end{array}$$

where ${𝐮}_{j}(s)$ is the output of primary capsule *j* for input *s*, and ${𝐖}_{ij}$ is a learned linear transformation. Dynamic routing maintains real-valued routing logits $b_{ij}$ and coupling coefficients $c_{ij}^{\;s}$ defined by a softmax over *i* for each fixed *j*:


$$\begin{array}{c} c_{ij}^{\;s}=\frac{\exp(b_{ij})}{\sum_{i^{\prime }=1}^{T}\exp(b_{i^{\prime }j})}. \end{array}$$ (13)

The type capsule input and output are computed as


$$\begin{array}{c} {𝐬}_{i}(s)=\sum_{j=1}^{J}c_{ij}^{\;s}\;{\hat{𝐮}}_{i|j}(s),\;{𝐯}_{i}(s)=squash({𝐬}_{i}(s)), \end{array}$$

where squash(·) is the standard nonlinearity ensuring $\left\|{𝐯}_{i}(s)\|\in (0,1\right)$.

**(1) Normalization**. For any fixed *j*, the right-hand side of (13) is the standard softmax distribution over i∈{1,...,T}. Therefore,


$$\begin{array}{c} \sum_{i=1}^{T}c_{ij}^{\;s}=\sum_{i=1}^{T}\frac{\exp(b_{ij})}{\sum_{i^{\prime }=1}^{T}\exp(b_{i^{\prime }j})}=\frac{\sum_{i=1}^{T}\exp(b_{ij})}{\sum_{i^{\prime }=1}^{T}\exp(b_{i^{\prime }j})}=1. \end{array}$$

Hence the coupling coefficients from a fixed primary capsule *j* form a probability distribution over type capsules.

**(2) Preference for Alignment.** Dynamic routing updates the logits $b_{ij}$ using an *agreement* term. A common and standard update rule is


$$\begin{array}{c} b_{ij}\leftarrow b_{ij}+a_{ij}(s),\;\text{where}\;a_{ij}(s)=\left\langle {\hat{𝐮}}_{i|j}(s),{𝐯}_{i}(s)\right\rangle , \end{array}$$ (14)

i.e., the scalar product between the prediction vector from capsule *j* to capsule *i* and the current output of capsule *i*. Consider two type capsules *i* and $i^{\prime }$ for a fixed *j*. If the agreement satisfies


$$\begin{array}{c} a_{ij}(s)>a_{i^{\prime }j}(s), \end{array}$$

then after the update (14), we have $b_{ij}$ increased by a larger amount than $b_{i^{\prime }j}$. Since $c_{ij}^{\;s}$ is a strictly increasing function of $b_{ij}$ when other logits are fixed (as a property of the softmax), it follows that the updated coupling coefficient to capsule *i* becomes larger relative to that of capsule $i^{\prime }$. More formally, for fixed *j*, define the softmax map


$$\begin{array}{c} \phi_{j}:{\mathbb{R}}^{T}\to (0,1{)}^{T},\;\phi_{j}({𝐛}_{\cdot j}{)}_{i}=\frac{\exp(b_{ij})}{\sum_{i^{\prime }}\exp(b_{i^{\prime }j})}. \end{array}$$

This map is componentwise monotone: if $b_{ij}$ increases while all other $b_{i^{\prime }j}$ remain fixed, then $\phi_{j}({𝐛}_{\cdot j}{)}_{i}$ increases. Therefore, repeated routing iterations amplify couplings toward those type capsules that maintain higher agreement values $a_{ij}(s)$.

In particular, when ${\hat{𝐮}}_{i|j}(s)$ is well aligned with ${𝐯}_{i}(s)$, the inner product in (14) is large, causing $b_{ij}$ and thus $c_{ij}^{\;s}$ to increase, meaning that primary capsule *j* assigns more of its output to explaining type capsule *i*. This establishes the stated preference-for-alignment behavior. □

An important implication of Proposition 1 is that, for each cell type *t*, there tends to exist at least one primary capsule *j* that specializes in capturing features of that type. Empirically, one can identify for each type capsule *i* the primary capsule *j* with the largest total coupling $c_{ij}$ (averaged over that type’s samples). We denote this capsule as $j^{\left(i\right)}=\text{arg}\max_{j}c_{ij}$ and term it a dedicated capsule for cell type *i*. In our experiments, we indeed observe a roughly one-to-one alignment between cell types and specific primary capsules (see, e.g., primary capsule 9 for cell type “CD16 + Mono” in Fig 1). This alignment justifies restricting attention, for each type *i*, to the primary capsule *j*^(*i*)^ that carries the strongest signal for that type when identifying marker genes.

We now formalize the procedure for marker gene identification and establish its statistical validity. Intuitively, if gene $g_{k}$ is a bona fide marker for cell type *i*, then feeding $g_{k}$ (in isolation) into the network should strongly activate the pathway from some primary capsule (ideally $j^{*}(i)$) to type capsule *i*, resulting in an unusually large coupling coefficient $c_{ij}^{k}$. By contrast, for a gene with no specific association to type *i*, the coupling $c_{ij}^{k}$ should fluctuate around some baseline (background noise level) and not produce extreme values. We capture this intuition by a hypothesis testing framework: treat each gene’s coupling $c_{ij}^{k}$ as a test statistic for the null hypothesis “gene $g_{k}$ is not a specific marker for type *i*.”

To proceed, we assume that the null distribution of the coupling coefficients (for non-marker genes) is approximately Gaussian. Empirically, the histogram of $c_{ij}^{k}:k=1,...,K$ often resembles a normal distribution centered around a mean, with a heavy tail on the right (corresponding to a few genes with exceptionally high coupling, see Fig 1, discussed later). Let $\mu_{ij}$ and $\sigma_{ij}$ be the mean and standard deviation of $c_{ij}^{k}$ across all genes for a given (*i*,*j*) combination. We define a one-tailed *p*-value for each gene $g_{k}$ with respect to type *i* (and its dedicated capsule *j*^(*i*)^) as


$$\begin{array}{c} p_{i}^{k}=\mathbb{P}\;\left\{Z>c_{ij^{*}(i)}^{k}\right\} \end{array}$$ (15)

where $Z\sim 𝒩(\mu_{i,j^{(}i)},,\sigma_{i,j^{(}i)}^{2})$ is a Gaussian random variable fitted to the null coupling distribution (genes in the middle of the distribution) for capsule *j*^(*i*)^. In other words, $p_{i}^{k}$ is the right-tail probability (extreme-value significance) of the observed coupling $c_{i,j^{*}(i)}^{k}$. Genes with exceptionally large coupling to type *i* will thus attain very small *p*-values.

Given these *p*-values for all genes, we employ the Benjamini–Hochberg (BH) procedure to identify significant marker genes while controlling the false discovery rate (FDR) at a chosen level α (we use α=0.05 by convention). Let $p^{\left(1\right)}i\leq p^{\left(2\right)}i\leq \cdots \leq p^{\left(K\right)}i$ be the sorted *p*-values for type *i*. The BH procedure finds the largest index *L* such that $p^{\left(L\right)}\leq \frac{L}{K}\;\alpha$ All genes corresponding to *p*-values $p^{\left(1\right)}i,...,p^{\left(L\right)}i$ are then declared as significant markers for cell type *i*. We denote the resulting set of marker genes by


$$\begin{array}{c} CM_{i}=\left\{g_{k}\;|\;p_{i}^{k}\leq p_{i}^{\left(L\right)}\right\} \end{array}$$ (16)

**Theorem 1** (Marker gene identification and FDR control). *Fix a cell type i and its selected primary capsule* $j^{*}(i)$*. For each gene* $g_{k}$*, let* $c_{ij^{*}(i)}^{k}$ *be the gene-specific coupling coefficient defined in (12). Let* $p_{i}^{k}$ *be the corresponding one-sided p-value defined by*


$$\begin{array}{c} p_{i}^{k}=\mathbb{P}\;\left\{Z>c_{ij^{*}(i)}^{k}\right\},\;Z\sim 𝒩\;\left(\mu_{ij^{*}(i)},\;\sigma_{ij^{*}(i)}^{2}\right), \end{array}$$

*and suppose that under the null hypothesis H*_*0,k*_ *(“*$g_{k}$ *is not a marker for type i”),* $p_{i}^{k}$ *is valid, i.e.,*


$$\begin{array}{c} \mathbb{P}(p_{i}^{k}\leq u)\leq u\;\text{for all }u\in [0,1]. \end{array}$$ (17)

*Apply the Benjamini–Hochberg (BH) procedure at level* α∈(0,1) *to the K p-values* $\{p_{i}^{k}{\}}_{k=1}^{K}$ *and let* $CM_{i}$ *be the resulting rejection set (marker set) as in (16). If the null p-values are independent (or satisfy the standard PRDS condition), then the false discovery rate satisfies*


$$\begin{array}{c} FDR\;=\;𝔼\;\left[\frac{V}{\max(R,1)}\right]\leq \alpha , \end{array}$$

*where* $R=|CM_{i}|$ *is the number of selected genes and V is the number of false selections. Moreover, if there exists a separation margin* Δ>0 *such that the coupling statistic for true marker genes lies strictly in the right tail of the null distribution, then the probability that a true marker gene is included in* $CM_{i}$ *converges to 1 as per-type sample sizes grow.*

*Proof.* We prove (A) FDR control under BH and (B) asymptotic detection of true markers. The FDR-control statement follows the standard Benjamini–Hochberg theory under independent or PRDS null p-values. We include the theorem for self-contained presentation. The novel aspect in the present work is the construction of capsule-coupling-based gene-specific statistics and their use for marker-gene identification within RareCapsNet.

#### (A) FDR control.

Fix the type *i* and write $p_{k}:=p_{i}^{k}$ for simplicity. Let ${ℋ}_{0}$ be the set of true null hypotheses (non-marker genes for type *i*), and let $m_{0}=|{ℋ}_{0}|$. Let $p_{\left(1\right)}\leq \cdots \leq p_{\left(K\right)}$ be the ordered p-values and define the BH threshold index


$$\begin{array}{c} L=\max\left\{\;\ell \in \{1,...,K\}\;|\;p_{\left(\ell \right)}\leq \frac{\ell }{K}\alpha \right\}, \end{array}$$

with the convention that *L* = 0 if the set is empty. The BH rejection set is


$$\begin{array}{c} ℛ=\{k:p_{k}\leq p_{\left(L\right)}\}, \end{array}$$

so R=|ℛ|=L and $CM_{i}=ℛ$. Let $V=|ℛ\cap {ℋ}_{0}|$ be the number of false discoveries.

To bound FDR=𝔼[V/max(R,1)], we use the classical BH argument. For each null index $k\in {ℋ}_{0}$, define the indicator of rejection $1\{p_{k}\leq p_{\left(L\right)}\}$. Then


$$\begin{array}{c} V=\sum_{k\in {ℋ}_{0}}1\{p_{k}\leq p_{\left(L\right)}\}. \end{array}$$

Hence, using linearity of expectation,


$$\begin{array}{c} FDR=𝔼\;\left[\frac{V}{\max(R,1)}\right]=\sum_{k\in {ℋ}_{0}}𝔼\;\left[\frac{1\{p_{k}\leq p_{\left(L\right)}\}}{\max(R,1)}\right]. \end{array}$$ (18)

We now condition on all p-values except $p_{k}$. Let ${𝐩}_{-k}$ denote the vector of p-values excluding $p_{k}$. Define $R_{k}(u)$ as the number of BH rejections that would occur if we set $p_{k}=u$ and keep ${𝐩}_{-k}$ fixed, i.e., apply BH to $\left(u,{𝐩}_{-k}\right)$. Under independence (or PRDS), one can show that the event $\left\{p_{k}\leq p_{\left(L\right)}\right\}$ implies $p_{k}\leq \alpha R/K$ and that *R* is nonincreasing in $p_{k}$. Therefore, on the event that $p_{k}$ is rejected and R≥1,


$$\begin{array}{c} \frac{1}{R}\leq \frac{\alpha }{K\;p_{k}}. \end{array}$$

Using this inequality inside (18) yields


$$\begin{array}{c} 𝔼\;\left[\frac{1\{p_{k}\leq p_{\left(L\right)}\}}{\max(R,1)}\right]\leq 𝔼\;\left[1\{p_{k}\leq p_{\left(L\right)}\}\frac{\alpha }{K\;p_{k}}\right]. \end{array}$$ (19)

Now apply the tower property conditioning on ${𝐩}_{-k}$:


$$\begin{array}{c} 𝔼\;\left[1\{p_{k}\leq p_{\left(L\right)}\}\frac{1}{p_{k}}\right]=𝔼\;\left[𝔼\;\left[1\{p_{k}\leq p_{\left(L\right)}\}\frac{1}{p_{k}}\;|\;{𝐩}_{-k}\right]\right]. \end{array}$$

For fixed ${𝐩}_{-k}$, the event $\left\{p_{k}\leq p_{\left(L\right)}\right\}$ is of the form $\left\{p_{k}\leq \tau \right\}$ for some data-dependent threshold $\tau =\tau ({𝐩}_{-k})\in [0,1]$ (this is a standard property of step-up procedures). Thus,


$$\begin{array}{c} 𝔼\;\left[1\{p_{k}\leq \tau \}\frac{1}{p_{k}}\;|\;{𝐩}_{-k}\right]=\int_{0}^{\tau }\frac{1}{u}\;dF_{k}(u), \end{array}$$

where $F_{k}(u)=\mathbb{P}(p_{k}\leq u|{𝐩}_{-k})$. Under the null, $p_{k}$ is valid and (under independence) uniform, so $F_{k}(u)=u$ and $dF_{k}(u)=du$. Hence the integral becomes


$$\begin{array}{c} \int_{0}^{\tau }\frac{1}{u}\;du=\log(\tau )-\log(0), \end{array}$$

which is improper; the classical BH proof avoids this route by using a sharper argument:


$$\begin{array}{c} 𝔼\;\left[\frac{1\{p_{k}\leq \alpha R/K\}}{R}\right]\leq \frac{\alpha }{K}. \end{array}$$

This inequality holds under independence (and extends under PRDS) and is the key lemma in the BH proof. Applying it to each $k\in {ℋ}_{0}$ and summing yields


$$\begin{array}{c} FDR=\sum_{k\in {ℋ}_{0}}𝔼\;\left[\frac{1\{p_{k}\leq p_{\left(L\right)}\}}{\max(R,1)}\right]\leq \sum_{k\in {ℋ}_{0}}\frac{\alpha }{K}=\frac{m_{0}}{K}\alpha \leq \alpha . \end{array}$$

Thus BH controls the FDR at level α.

#### (B) Asymptotic detection of true marker genes.

Let $g_{k}$ be a true marker gene for type *i*. Consider the stratified estimator


$$\begin{array}{c} c_{ij^{*}(i)}^{k}=\frac{1}{T}\sum_{t=1}^{T}\frac{1}{N(t)}\sum_{\begin{array}{c} s\in S\\ t(s)=t \end{array}}c_{ij^{*}(i)}^{k,s}. \end{array}$$

Assume finite second moments and define the type-conditional mean


$$\begin{array}{c} \mu_{t}^{k}:=𝔼\;\left[c_{ij^{*}(i)}^{k,s}|t(s)=t\right]. \end{array}$$

By the strong law of large numbers, for each *t*,


$$\begin{array}{c} \frac{1}{N(t)}\sum_{\begin{array}{c} s\in S\\ t(s)=t \end{array}}c_{ij^{*}(i)}^{k,s}\;\overset{a.s.\;}{\to }\;\mu_{t}^{k},\;\text{as }N(t)\to \infty , \end{array}$$

and therefore,


$$\begin{array}{c} c_{ij^{*}(i)}^{k}\;\overset{a.s.\;}{\to }\;\frac{1}{T}\sum_{t=1}^{T}\mu_{t}^{k}. \end{array}$$

Assume a strict separation condition: there exists Δ>0 such that


$$\begin{array}{c} \mu_{i}^{k}\geq \mu_{t}^{k}+\Delta \;\text{for all }t\neq i, \end{array}$$

which captures that gene $g_{k}$ produces systematically higher routing agreement toward type capsule *i* (through capsule $j^{*}(i)$) for cells of type *i*. Then the limit $\frac{1}{T}\sum_{t}\mu_{t}^{k}$ lies strictly to the right of the null mean used to compute $p_{k}$, implying that $p_{k}\to 0$ as sample sizes grow, because the right-tail probability of a Gaussian decays to 0 as the observed statistic moves deeper into the upper tail. Consequently, for sufficiently large sample sizes, $p_{k}$ will be among the smallest p-values and will pass the BH threshold with probability approaching 1. Hence a true marker gene is selected with probability tending to 1. □

The above theoretical framework demonstrates that our coupling-based gene attribution method is grounded in sound principles. Coupling coefficients provide an interpretable measure of gene influence on capsule–type associations (Proposition 1), and by analyzing their distribution we can identify marker genes with statistical confidence (Theorem 1). We next describe the practical algorithms that implement these ideas in the RareCapsNet network.

### Algorithm for Computation of Gene Specific Coupling Coefficients for rare cells

**Algorithm 1 Compute Gene Specific Coupling Coefficients**

**Require:** Trained marker capsule network, training dataset *S*

**Ensure:** Coupling coefficients $c_{ij}^{k}$ for each gene $g_{k}$, primary capsule *j*, and type capsule *i*

1: **for** each gene $g_{k}$, k=1,...,K **do**

2:  **for** each sample s∈S **do**

3:   Construct masked input vector $v^{k,s}$ by retaining only expression value of $g_{k}$

4:   Run $v^{k,s}$ through the trained network

5:   Extract coupling coefficients $c_{ij}^{k,s}$ between primary capsule *j* and type capsule *i*

6:  **end for**

7: **end for**

8: **for** each type capsule i=1,...,I and primary capsule j=1,...,J **do**

9:  **for** each gene $g_{k}$ **do**

10:     Compute average: $c_{ij}^{k}\leftarrow \frac{1}{\left|S_{i}\right|}\sum_{s:t(s)=i}c_{ij}^{k,s}$ {$S_{i}$ is the set of samples with cell type *i*}

11:  **end for**

12: **end for**

13: **return** $c_{ij}^{k}$ for all *k*, *i*, and *j*

Let *k* = 1,...,*K* refer to genes $g_{k}$, *t* = 1,...,*T* to cell types, *j* = 1,...,*J* to primary capsules, and *i* = 1,...,*I t*o type capsules. Note that although *T* = *I* we need to distinguish between types of single cells *t* and type capsules *i* in the following, which explains the differen*t* indices. Let further *S* be the training single cell samples. Each single cell s∈S corresponds to a *K*-dimensional real valued vector, where entries *s*[*k*], *k* = 1,...,*K* correspond to the expression of gene $g_{k}$ in single cell *s*. Training data are labeled by cell types, we refer to *t*(*s*) as the type of single cell *s*. Let N(t)=#{s∈S∣t(s)=t} be the number of single cell samples s∈S for which *t*(*s*)=*t*.

According to the setup of the RareCapsNet architecture, *J* = 32 for all data sets. The number of cell types *T* (and hence *I* = *T*) can vary with *T* taking values between 11 and 15. Equally, the number of genes *K* supported by the particular data set can vary. In the experiments in which the algorithm here is used, we make use of data set 1 (mRNA + protein expression; CITE-seq [22]). According to this data set, *T* = *I* = 13 and *K* = 2000, see also the detailed specifications of the data sets in the Supplement.

In the following we outline an algorithm that, when provided with the trained MarkerCapsule network architecture and the training samples *S*, will output gene specific coupling coefficients $c_{ij}^{k}$. These $c_{ij}^{k}$ indicate how strongly gene $g_{k}$ contributes to activating the link from primary capsule *j* to cell type capsule *i*.

The algorithm proceeds according to the following step

1. For each training sample s∈S and each gene $g_{k},k=1,...,K$, consider $s^{k}$ defined by
  $$\begin{array}{c} s^{k}[j]=\left\{ \begin{array}{cc} s[k] & j=k\\ 0 & \text{otherwise} \end{array}\right. \end{array}$$ (20)
  That is, $s^{k}$ keeps *s*[*k*] from the original input of single cell *s* and has all other entries masked, that is, set to zero.
2. For each combination (*s*,*k*) of single cell *s* and gene $g_{k}$, we pass the masked vector $s^{k}$ through the trained RareCapsNet network. In the present experiments, no additional auxiliary data were used, and therefore $s^{k}$ itself is provided as the network input. If future extensions include auxiliary cell-level covariates or additional modalities, such data should first be preprocessed and aligned with the corresponding cell *s*, and then combined with $s^{k}$ according to the specified input representation before being passed to the network.
3. Running $s^{k}$ through the network includes executing the dynamic routing procedure, hence yields coupling coefficients $c_{ij}^{s,k}$ for all *i* = 1,...,*I*, *j* = 1,...,*J*
4. One averages the resulting $c_{ij}^{s,k}$ across samples assigning equal weight to each cell type *t*:
  $$\begin{array}{c} c_{ij}^{k}=\frac{1}{T}\sum_{t=1}^{T}(\frac{1}{N(t)}\sum_{s:t(s)=t}c_{ij}^{k,s}) \end{array}$$ (21)
5. One outputs $c_{ij}^{k}$ as the desired gene specific coupling coefficients.

### Algorithm for computing marker genes through rare capsules

Given a particular cell type *i*, one determines the primary capsule *j* that yields the largest coupling coefficient $c_{ij}$. Note that if several primary capsules *j* yield sufficiently large $c_{ij}$, one can extend the analysis on all such *j*; for the sake of an analysis that gives rise to an unbiased comparison across cell types, we restrict ourselves to the primary capsule *j* that yields the larges $c_{ij}$ for each *i*.

One then determines the gene specific coupling coupling coefficients $c_{ij}^{k}$ for all genes $g_{k},k=1,...,K$, as per the algorithm described in Subsection. Subsequently, one determines the mean and the standard deviation of the $c_{ij}^{k},k=1,...,K$; as can be seen in Fig 1 (for the combination of primary capsule 9 and cell type ’CD16 + Mono’, which exemplifies the situation for all selected combinations *j* and *i*), the empirical distribution of the $c_{ij}^{k}$ roughly follows a Gaussian distribution, with the exception of an heavy tail towards the right. The idea is to collect all genes that give rise to this heavy tail.

To do so in a sound way, we determine p-values $p_{ij}^{k},k=1,...,K$ reflecting tail probabilities with respect to the Gaussian distribution the usual way. Subsequently, we sort the resulting $p_{ij}^{k}$ in ascending order (where the smallest $p_{ij}^{k}$ corresponds with the largest $c_{ij}^{k}$) yielding


$$\begin{array}{c} p_{ij}^{k_{1}}\leq p_{ij}^{k_{2}}\leq ...\leq p_{ij}^{k_{K}} \end{array}$$

and determines


$$\begin{array}{c} L=\max\left\{\;l\in \{1,...,K\}\;|\;p_{ij}^{k_{l}}\leq \frac{l}{K}\alpha \right\}. \end{array}$$

that is the largest *l* for which $p_{ij}^{k_{l}}\leq \frac{l}{K}\alpha$, where α reflects the significance level at which one operates. Here, α=0.05, which follows standard conventions. Overall, this procedure reflects the Benjamini-Hochberg procedure, which accounts for the necessary corrections due to multiple hypothesis testing [31–33].

The set of marker genes $CM_{i}$ for cell type *i* is then defined to be


$$\begin{array}{c} CM_{i}=g_{k}|p_{ij}^{k}\leq p_{ij}^{k_{L}} \end{array}$$ (22)

## Conclusions

In this work, we presented **RareCapsNet**, an interpretable capsule network–based framework for robust identification of rare cell populations from large-scale single-cell RNA sequencing data. By leveraging the dynamic routing mechanism of capsule networks, RareCapsNet explicitly models part–whole relationships between genes, primary capsules, and type capsules, enabling reliable detection of rare cellular identities even under extreme class imbalance and high dropout conditions, where conventional clustering-based approaches often fail.

A key strength of RareCapsNet lies in its interpretability. Through gene-specific coupling coefficients and statistically principled false discovery rate control, the framework not only detects rare cells but also identifies biologically meaningful marker genes associated with distinct capsule activations. Theoretical analysis establishes conditions for identifiability of rare cell types, robustness to class imbalance through routing normalization, and convergence of the routing procedure, thereby providing a sound mathematical basis for the observed empirical performance.

To evaluate generalizability, we performed cross-dataset transfer experiments, where RareCapsNet trained on one dataset was used to infer rare cell identities in a related but unseen dataset. The results suggest that capsule routing activations for rare subpopulations can remain informative across related datasets when source and target data are represented in a shared gene space. However, this observation should be interpreted within the evaluated transfer settings, and broader cross-platform generalization will require further validation.

Although the present implementation is supervised and does not perform fully open-set discovery of unseen cell types, RareCapsNet offers a promising foundation for future extensions. Semi-supervised and weakly supervised formulations may allow the model to incorporate partial, noisy, or incomplete annotations, thereby improving usability in real-world single-cell studies where fully curated labels are often unavailable. Uncertainty-aware rejection thresholds or open-set capsule learning may further help distinguish known rare cell types from genuinely novel cellular populations.

Future extensions may also include multimodal and spatial adaptations of RareCapsNet. For example, transcriptomic features may be integrated with protein-level measurements from CITE-seq or spatial coordinates from spatial transcriptomics through capsule-level fusion, enabling improved resolution of rare cell identity and tissue context. In addition, model-compression strategies, sparse routing, and pruning-based optimization may improve scalability for ultra-large tissue atlases and reduce computational overhead during inference and gene-attribution analysis.

In conclusion, RareCapsNet offers a scalable, interpretable, and theoretically grounded solution for rare cell discovery. It advances methodological rigor in rare-cell detection while unlocking biological insights through capsule-level gene attribution and cross-study generalization, marking a step forward in the analysis of high-resolution single-cell transcriptomic data.

## Supporting information

S1 Text Supplementary methods, analyses, figures, and tables supporting the RareCapsNet study. (PDF)
