# Supplementary material for: RareCapsNet: An explainable capsule network enables robust discovery of rare cell populations from large-scale single-cell transcriptomics
Source: PLoS Comput Biol. 2026 Aug 10;22(8):e1013962. doi: 10.1371/journal.pcbi.1013962 (PMC13472507; doi:10.1371/journal.pcbi.1013962)
Supplement: Supplementary file 1 [file pcbi.1013962.s001.pdf]

RareCapsNet: An explainable capsule networks enable robust discovery of rare cell populations from large-scale single-cell transcriptomics

Sumanta Ray[1,*], Snehalika Lall[2]

[1]Data Science, West Bengal National University of Juridical Sciences, Kolkata, West Bengal, India.
[2]Human Genetics Unit, Indian Statistical Institute, Kolkata, West Bengal, India.

Additional details on deep learning-based baseline comparisons

To complement the main Results section, we provide additional details on the comparison between RareCapsNet and modern deep learning-based single-cell methods. The selected baselines include scVI [1], scANVI [2], CellAssign [3], and ItClust [4], which represent widely used deep generative, semi-supervised, marker-informed, and transfer-learning-based approaches for single-cell analysis.

All methods were evaluated using the same preprocessed expression matrices used for RareCapsNet. For each dataset, cells were normalized, log-transformed, and represented using the selected highly variable genes. For scVI and scANVI, the learned latent representations were used for downstream rare-cell classification. For CellAssign and ItClust, the predicted cell-type assignments were compared with the reference labels. Rare-cell detection performance was quantified using the F1-score of the rare cell population, in order to maintain consistency with the evaluation metric used in the main manuscript.

Table A: **Extended comparison of RareCapsNet with classical and deep learning-based single-cell methods.** F1-scores are reported for rare-cell detection across PBMC68k, CBMC, Zeisel mouse brain, and a cross-batch transfer setting. Deep learning baselines include scVI, scANVI, CellAssign, and ItClust.

Dataset / Setting	RareCapsNet	RaceID	GiniClust	FiRE	scVI	scANVI	CellAssign	ItClust
PBMC68k	**0.80**	0.72	0.67	0.78	0.76	0.78	0.74	0.79
CBMC	**0.81**	0.68	0.62	0.74	0.74	0.75	0.72	0.77
Zeisel brain	**0.84**	0.69	0.65	0.76	0.70	0.71	0.68	0.74
Cross-batch transfer	**0.78**	0.62	0.60	0.73	0.69	0.72	0.70	0.75

Table A shows that the deep learning-based methods generally outperform classical rare-cell detection tools, especially in the PBMC68k and cross-batch transfer settings. However, RareCapsNet consistently achieves the highest F1-score across all evaluated datasets. This suggests that the capsule-routing mechanism provides additional benefit for rare-cell-specific representation learning, particularly when rare populations are small or when data are transferred across batches.

Unlike general-purpose latent representation models, RareCapsNet explicitly links prediction to capsule-level routing and gene-specific coupling coefficients. Therefore, the performance improve-

ment is accompanied by an interpretable mechanism for identifying the gene features associated with rare-cell populations.

Runtime, memory usage, and scalability

To assess the computational cost of RareCapsNet, we compared its approximate training time and peak GPU memory consumption with representative deep learning-based single-cell analysis methods, including scVI, scANVI, CellAssign, and ItClust. All models were benchmarked on a PBMC68k-sized dataset using an NVIDIA RTX 3090 GPU with 24 GB memory under comparable preprocessing and training settings.

Table B: **Runtime and GPU memory usage comparison across methods.** Approximate training time and peak GPU memory usage are reported for RareCapsNet and four deep learning baselines evaluated on a PBMC68k-sized dataset using an NVIDIA RTX 3090 GPU with 24 GB memory.

**Method**	**Runtime (min)**	**Peak GPU Memory (GB)**
RareCapsNet	25	8.5
scVI	20	6.2
scANVI	16	6.8
CellAssign	20	7.1
ItClust	15	7.4

Table B shows that RareCapsNet requires moderately higher runtime and GPU memory than the compared deep learning baselines. This increase is expected because RareCapsNet performs iterative dynamic routing between primary capsules and type capsules, whereas methods such as scVI and scANVI rely on simpler variational encoder-decoder structures. The additional computational cost is therefore associated with the capsule routing mechanism that enables capsule-level interpretability and gene-level attribution.

RareCapsNet required approximately 25 minutes to train and consumed 8.5 GB of peak GPU memory on the PBMC68k-sized benchmark. Although this is higher than the runtime and memory usage of scVI, scANVI, CellAssign, and ItClust in our benchmark, the computational requirement remains practical for datasets containing tens of thousands of cells. In our experiments, the number of primary capsules and routing iterations were fixed across datasets, and the dominant training cost scaled approximately linearly with the number of cells and input genes after highly variable gene selection.

We also note that the post hoc gene-attribution step is more computationally demanding than model training because it requires repeated forward passes under masked gene inputs. However, this analysis is performed after training and can be parallelized across genes or restricted to a selected set of highly variable or candidate marker genes. Future implementation-level improvements may include sparse routing, shared transformation matrices, approximate routing updates, and GPU-parallelized gene attribution to improve scalability for atlas-scale single-cell datasets.

Cross-Dataset Generalization

. For cross-dataset transfer experiments, source and target datasets were first independently normalized and log-transformed. Highly variable genes were selected for each dataset, and only the genes common to both the source and target datasets were retained. The resulting intersected gene matrices were reordered to ensure identical feature ordering before applying the trained RareCapsNet model to the target dataset. No additional transfer layer, gene mapping model, or domain-adaptation procedure was used. This design allowed us to test whether capsule activations learned on one dataset remained informative in another dataset under a shared-gene representation. We note that transfer across datasets with substantially non-overlapping gene panels remains an important limitation and a direction for future work.

Extended biological validation of capsule-associated marker genes

To complement the main-text marker-gene validation analysis, we provide additional details on how the biological relevance of capsule-associated genes was assessed. For each rare cell population, genes were ranked according to the gene-specific coupling coefficient derived from the selected primary capsule and corresponding type capsule. The top-ranked genes were then evaluated using three complementary strategies: overlap with public marker resources, functional enrichment analysis, and literature-guided interpretation of highly ranked non-canonical genes.

First, we compared RareCapsNet-derived capsule-associated genes with public marker resources, including CellMarker, PanglaoDB, and curated PBMC or brain cell-type annotations. This analysis confirmed that several highly ranked capsule-associated genes overlapped with established rare-cell markers. For example, pDC-associated capsules recovered *CLEC4C*, *LILRA4*, *TCF4*, and *IRF7*; dendritic-cell-associated capsules recovered *FCER1A*, *CST3*, and *HLA-DRA*; and megakaryocyte-associated capsules recovered *PF4*, *PPBP*, *ITGA2B*, and *GP9*.

Second, we performed functional enrichment analysis using GO Biological Process and KEGG pathway annotations. The enriched terms were consistent with the expected biological functions of the corresponding rare cell populations. pDC-associated genes were enriched for type I interferon signaling, antiviral defense response, and innate immune activation. Dendritic-cell-associated genes were enriched for antigen processing and presentation, leukocyte activation, and immune response regulation. Megakaryocyte-associated genes were enriched for platelet activation, platelet degranulation, coagulation, and wound-healing-related processes.

Third, genes that ranked highly by RareCapsNet but were not included in canonical marker lists were examined as putative capsule-associated genes. These genes were interpreted in the context of enriched biological processes and available literature rather than being claimed as definitive novel biomarkers. This conservative interpretation strengthens the biological validity of the capsule-based explanations while avoiding overstatement of marker novelty.

Table C: **Extended biological validation of capsule-associated genes.** Representative top-ranked genes identified by RareCapsNet are shown together with known marker overlap and enriched biological functions.

Rare cell population	Top capsule-associated genes	Marker database overlap	Representative enriched functions
pDCs	*CLEC4C*, *LILRA4*, *TCF4*, *IRF7*	CellMarker / PanglaoDB / PBMC markers	Type I interferon signaling; antiviral response; innate immunity
Dendritic cells	*FCER1A*, *CST3*, *HLA-DRA*, *LST1*	CellMarker / PanglaoDB / PBMC markers	Antigen processing and presentation; leukocyte activation; immune regulation
Megakaryocytes	*PF4*, *PPBP*, *ITGA2B*, *GP9*	CellMarker / PanglaoDB / blood-cell markers	Platelet activation; platelet degranulation; coagulation; wound healing
Neurogliaform/interneuron-like cells	*RELN*, *NDNF*, *GAD1*, *GAD2*	Brain cell-type marker annotations	GABAergic synaptic signaling; neuronal differentiation; interneuron identity

Ablation Study – Effect of Capsule Parameters

To evaluate the influence of capsule-network design choices on rare-cell detection, we performed an ablation study under simulated rare-cell settings. In addition to overall classification performance, we assessed the model using accuracy, sensitivity/recall, and ROC-AUC. Accuracy measures the overall correctness of classification, sensitivity/recall evaluates the ability of the model to recover rare-cell populations, and ROC-AUC measures the discrimination ability of the model across different decision thresholds. Since rare-cell detection is strongly affected by class imbalance, sensitivity/recall is particularly important because a model may achieve high accuracy by correctly classifying majority cells while still failing to detect rare populations.

Table D summarizes the comparative performance of RareCapsNet and four baseline rare-cell detection methods under the two-class simulated rare-cell settings S1–S2. RareCapsNet achieved the highest overall performance, with an accuracy of 0.952, sensitivity/recall of 0.865, and ROC-AUC of 0.956. Among the baseline methods, FiRE and CellSIUS showed relatively competitive performance, whereas GiniClust and RaceID showed comparatively lower sensitivity/recall. This indicates that the capsule-routing mechanism in RareCapsNet improves the recovery of rare-cell populations under strong class imbalance while maintaining high overall classification accuracy.

Table D: Comparative performance of RareCapsNet and baseline methods on two-class simulated rare-cell datasets. Values are reported as average performance across S1 and S2 simulation settings. Sensitivity/recall corresponds to recall for the rare-cell class. ROC-AUC denotes the area under the receiver operating characteristic curve.

Method	Accuracy	Sensitivity/Recall	ROC-AUC
RareCapsNet	0.952	0.865	0.956
GiniClust	0.919	0.715	0.872
FiRE	0.936	0.791	0.904
RaceID	0.931	0.747	0.889
CellSIUS	0.928	0.764	0.895

Table E reports the comparative performance under the more complex multi-class and multi-rare-cell simulation settings S3–S5. These settings are more challenging because the model must distinguish multiple cell populations, including more than one rare class. RareCapsNet again achieved the best overall performance, with an accuracy of 0.939, sensitivity/recall of 0.849, and ROC-AUC of 0.947. The improvement in sensitivity/recall is particularly important because it indicates that RareCapsNet retained stronger ability to detect minority populations even when several rare classes were present simultaneously. Among the baseline methods, FiRE and CellSIUS showed moderate performance, but their lower sensitivity/recall and ROC-AUC suggest that they were less effective in recovering rare classes than RareCapsNet. These findings indicate that the proposed capsule-based architecture is robust not only in binary rare-cell detection but also in more complex multi-class rare-cell scenarios.

Overall, the comparative simulation results show that RareCapsNet provides a favorable balance between overall classification accuracy and rare-cell sensitivity. The improvement is most evident in sensitivity/recall and ROC-AUC, which are more informative than accuracy alone in imbalanced rare-cell detection tasks. These results support the use of the selected capsule-network configuration in the main experiments and suggest that dynamic routing helps preserve rare-cell-specific

Table E: Comparative performance of RareCapsNet and baseline methods on multi-class simulated rare-cell datasets. Values are reported as average performance across S3, S4, and S5 simulation settings. Sensitivity/recall corresponds to macro-averaged recall across rare-cell classes. ROC-AUC was computed using one-vs-rest classification where applicable.

Method	Accuracy	Sensitivity/Recall	ROC-AUC
RareCapsNet	0.939	0.849	0.947
GiniClust	0.901	0.692	0.853
FiRE	0.914	0.756	0.889
RaceID	0.908	0.721	0.871
CellSIUS	0.912	0.738	0.882

transcriptomic signals that may be weakened in conventional clustering-based rare-cell detection approaches.

Public scRNA-seq Datasets

We evaluated RareCap on four public single-cell transcriptomic datasets to demonstrate its performance on real biological data. Below we provide a brief description of each dataset, including its origin, composition, and source accession:

Jurkat–293T mixed cell line dataset: This dataset consists of a mixture of two human cell lines – Jurkat (an immortalized T-lymphocyte leukemia cell line) and HEK 293T (a human embryonic kidney cell line). The Jurkat cell line, originally derived from the peripheral blood of a T-cell leukemia patient, is widely used to study T-cell signaling. In the mixed dataset, Jurkat cells were present as an artificial rare population (only ∼2.5% of ∼3,200 total cells) amid a majority of 293T cells. This extreme dilution of Jurkat cells provides a ground truth for rare cell detection (Jurkat vs. 293T). We obtained the 293T/Jurkat mixture data from a 10x Genomics single-cell experiment which is publicly available via the 10x Genomics data portal (originally used in rare-cell benchmarks by Zhang *et al.* and others). RareCap was able to successfully separate the minority Jurkat cells and identify their gene signatures in this challenging 2.5% scenario.

CBMC CITE-seq dataset (Cord Blood Mononuclear Cells): This is a multimodal single-cell dataset of ∼8,000 cord blood mononuclear cells profiled by CITE-seq (simultaneous measurement of single-cell transcriptomes and cell-surface protein levels) The data (GEO: GSE100866) were originally published by Stoeckius *et al.*, who profiled cord blood cells with 13 antibody-tagged surface markers. The CBMC population encompasses a broad range of immune cell types – T cells, B cells, NK cells, monocytes, dendritic cells, megakaryocytes, hematopoietic progenitors, etc.. Notably, some cell types are very rare in this dataset: for example, conventional dendritic cells (cDC), plasmacytoid dendritic cells (pDC), and megakaryocytes each constitute well under 1% of the cells (dozens of cells out of thousands). This dataset thus provides a realistic test for rare cell discovery in an annotated mixture. RareCap was trained on 80% of the CBMC data and successfully detected these rare immune subsets in the test portion, including the Mk, cDC, and pDC populations that standard analyses struggle to distinguish. The known biological context of cord blood (with a preponderance of monocytes and T cells, and very few dendritic cells) makes this a valuable benchmark for rare cell identification. The raw data are available from GEO under accession GSE100866, and processed versions can be accessed through the Seurat data

portal PBMC 68k dataset (Peripheral Blood Mononuclear Cells): The PBMC68k dataset contains 68,579 single PBMCs from a healthy human donor, sequenced using the 10x Genomics Chromium platform (3' UMI profiling). This dataset, originally released by Zheng *et al.*, is one of the largest early scRNA-seq demonstrations and is available via the 10x Genomics repository (also mirrored in GEO under accession GSE115189). The cells cover at least 11 distinct immune cell subtypes, including $CD8^+$ T cells, $CD4^+$ naive and memory T cells, B cells, NK cells, monocytes, and rare dendritic cell subsets. A known challenge in this dataset is the identification of the dendritic cells (particularly pDCs, which are exceedingly few, and cDCs) because they form only a tiny fraction of PBMCs (on the order of ~3% combined) and tend not to cluster distinctly with standard methods. We used RareCap to analyze the PBMC68k data (with cells' ground-truth labels from Zheng *et al.*) and found that it could isolate the dendritic cell subpopulations by capsule-based features, successfully boosting the detection of these rare cells. In particular, RareCap uncovered strong capsule activation signals associated with DCs, and recovered known DC marker genes (e.g. CLEC4C, LILRA4) as well as putative novel markers—underscoring its ability to work on large, complex mixtures.

Zeisel mouse brain cell dataset: This is a well-known single-cell RNA-seq dataset from the mouse nervous system, first published by Zeisel *et al.* (Science 2015). It profiles 3005 cells from the mouse somatosensory cortex and hippocampal CA1 region, captured using the Fluidigm C1/STRT-seq protocol. The Zeisel dataset includes a rich diversity of brain cell types: multiple neuronal subtypes (interneurons, pyramidal neurons) as well as non-neuronal cells (astrocytes, oligodendrocytes, microglia, etc.). Among these, a few cell types are quite rare – for example, neurogliaform cells (a type of GABAergic interneuron) and certain glial or ependymal cell subtypes were represented by very small numbers of cells. RareCap's evaluation on this dataset demonstrates that it can identify such rare neuronal subpopulations. By training on a portion of the Zeisel data, RareCap discovered specific primary capsule activations corresponding to the neurogliaform cells and other minor types, and highlighted marker genes like *Reln*, *Ptn*, and *Ndnf* that are known to be enriched in those cells. The dataset (GEO: GSE60361) thus provided a rigorous test in a complex tissue; RareCap achieved higher rare-cell detection accuracy (F1-score 0.88 for rare subtypes) compared to methods like RaceID, GiniClust, and CellSIUS on this benchmark.

Summary of Public Datasets Used in Evaluation

Table F: Key properties of the public scRNA-seq datasets used to evaluate RareCap.

Dataset	#Cells	#Genes	#Cell Types	Rare Cell Proportion
Jurkat–293T mixture	~3,200	~2,000	2	2.5% (Jurkat)
CBMC (CITE-seq)	~8,000	~2,000	13	<1% (Mk, pDC, cDC)
PBMC 68k	68,579	~20,000	≥11	~3% (cDC + pDC)
Zeisel brain	3,005	~19,972	9 major types	~1–2% (e.g., neurogliaform)

Datasets used for cross-dataset transfer analysis

We used four public single-cell RNA-seq datasets to evaluate the cross-dataset transfer behavior of RareCapsNet: Yan, Pollen, Darmanis, and CBMC. These datasets differ in tissue origin, sequencing

protocol, sample size, and cell-type composition, making them useful for assessing whether capsule activations learned from one dataset remain informative when evaluated on another dataset represented in a shared gene space.

Yan dataset. The Yan dataset consists of single-cell RNA-seq profiles from human preimplantation embryos and embryonic stem cells [5]. It includes cells from multiple early developmental stages, such as oocyte, zygote, 2-cell, 4-cell, 8-cell, morula, and blastocyst stages. This dataset provides a developmental single-cell setting with distinct cell-state labels and relatively small sample size.

Pollen dataset. The Pollen dataset contains low-coverage single-cell mRNA sequencing profiles from diverse cell types, including developing cerebral cortex-related cells and other cellular populations [6]. The dataset is commonly used to evaluate single-cell classification and clustering methods because it includes multiple annotated cell types and heterogeneous expression profiles.

Darmanis dataset. The Darmanis dataset profiles human brain transcriptomic diversity at the single-cell level [7]. It contains neuronal and non-neuronal cell populations from adult and fetal human brain samples. This dataset provides a biologically heterogeneous setting for evaluating transferability across neural single-cell data.

CBMC dataset. The CBMC dataset consists of cord blood mononuclear cells profiled using CITE-seq, which jointly measures transcriptomic and surface-protein information at single-cell resolution [8]. In this work, we used the transcriptomic component for RareCapsNet evaluation. The dataset contains diverse immune cell populations, including T cells, B cells, NK cells, monocytes, dendritic cells, megakaryocytes, and rare immune subsets.

For cross-dataset transfer experiments, each dataset was independently normalized and log-transformed. Highly variable genes were selected, and only the intersecting genes shared by the source and target datasets were retained. The resulting gene matrices were ordered identically before applying the trained RareCapsNet model to the target dataset.

References

[1] R. Lopez, J. Regier, M. B. Cole, M. I. Jordan, and N. Yosef, "Deep generative modeling for single-cell transcriptomics," *Nature Methods*, vol. 15, no. 12, pp. 1053–1058, 2018.

[2] C. Xu, R. Lopez, E. Mehlman, J. Regier, M. I. Jordan, and N. Yosef, "Probabilistic harmonization and annotation of single-cell transcriptomics data with deep generative models," *Molecular Systems Biology*, vol. 17, no. 1, p. e9620, 2021.

[3] A. W. Zhang, C. O'Flanagan, E. A. Chavez, J. L. P. Lim, N. Ceglia, A. McPherson, M. Wiens, P. Walters, T. Chan, B. Hewitson *et al.*, "Probabilistic cell-type assignment of single-cell RNA-seq for tumor microenvironment profiling," *Nature Methods*, vol. 16, no. 10, pp. 1007–1015, 2019.

[4] J. Hu, X. Li, G. Hu, Y. Lyu, K. Susztak, and M. Li, "Iterative transfer learning with neural network for clustering and cell type classification in single-cell RNA-seq analysis," *Nature Machine Intelligence*, vol. 2, no. 10, pp. 607–618, 2020.

[5] L. Yan, M. Yang, H. Guo, L. Yang, J. Wu, R. Li, P. Liu, Y. Lian, X. Zheng, J. Yan *et al.*, "Single-cell rna-seq profiling of human preimplantation embryos and embryonic stem cells," *Nature structural & molecular biology*, vol. 20, no. 9, p. 1131, 2013.

[6] A. A. Pollen, T. J. Nowakowski, J. Shuga, X. Wang, A. A. Leyrat, J. H. Lui, N. Li, L. Szpankowski, B. Fowler, P. Chen *et al.*, "Low-coverage single-cell mrna sequencing reveals cellular heterogeneity and activated signaling pathways in developing cerebral cortex," *Nature biotechnology*, vol. 32, no. 10, p. 1053, 2014.

[7] S. Darmanis, S. A. Sloan, Y. Zhang, M. Enge, C. Caneda, L. M. Shuer, M. G. H. Gephart, B. A. Barres, and S. R. Quake, "A survey of human brain transcriptome diversity at the single cell level," *Proceedings of the National Academy of Sciences*, vol. 112, no. 23, pp. 7285–7290, 2015.

[8] M. Stoeckius, C. Hafemeister, W. Stephenson, B. Houck-Loomis, P. K. Chattopadhyay, H. Swerdlow, R. Satija, and P. Smibert, "Simultaneous epitope and transcriptome measurement in single cells," *Nature Methods*, vol. 14, no. 9, pp. 865–868, 2017.
