# Supplementary material for: RareCapsNet: An explainable capsule network enables robust discovery of rare cell populations from large-scale single-cell transcriptomics
Source: PLoS Comput Biol. 2026 Aug 10;22(8):e1013962. doi: 10.1371/journal.pcbi.1013962 (PMC13472507; doi:10.1371/journal.pcbi.1013962)
Supplement: Supplementary file 2 [file pcbi.1013962.s004.pdf]

Answer to the Reviewer-1

General

This manuscript introduces RareCapsNet, a capsule network-based framework for the identification of rare cell populations from single-cell RNA-seq data. The model leverages dynamic routing to model part-whole relationships between genes, primary capsules, and cell-type capsules. However, several methodological inconsistencies, missing baselines, and reproducibility concerns need to be addressed before the manuscript can be accepted for publication.

Major

1. The comparison is limited to classical rare-cell tools (RaceID, GiniClust, FiRE). No deep learning-based baselines (e.g., scVI, scANVI, CellAssign, ItClust) are included. This is particularly concerning for the PBMC68k and cross-batch experiments, where deep generative models are now standard. It is recommended to add at least 2–3 modern DL-based methods to the benchmark.

Answer:

We thank the reviewer for this important and constructive suggestion. In response, we have added a new subsection in the Results section entitled **``Comparison with deep learning-based single-cell methods''**. In this subsection, we benchmark RareCapsNet against four widely used deep learning-based methods: **scVI, scANVI, CellAssign,** and **ItClust**, in addition to the classical rare-cell detection methods already considered.

The selected deep learning baselines represent complementary modeling strategies: scVI is a variational autoencoder-based generative model for single-cell transcriptomics, scANVI extends this framework for semi-supervised annotation and data harmonization, CellAssign is a probabilistic marker-informed cell-type assignment method, and ItClust is a neural network-based transfer learning method for clustering and cell-type classification. We evaluated these methods on PBMC68k, CBMC, Zeisel mouse brain, and a cross-batch transfer scenario using F1-score for rare-cell detection as the primary metric.

The results are now presented in the subsection **``Comparison with deep learning-based single-cell methods”** of the revised version of the manuscript (page no 10), with additional details retained in the Supplementary Material (subsection: **“Additional details on deep learning-based baseline comparisons”**). The expanded benchmark shows that deep learning baselines generally improve over classical rare-cell detection methods, however, RareCapsNet consistently achieves the highest F1-scores across all evaluated settings. The improvement is

particularly evident in the cross-batch transfer experiment, where RareCapsNet retains stronger rare-cell classification performance than scANVI and ItClust. We believe this addition substantially strengthens the evaluation and situates RareCapsNet more clearly within the current deep learning ecosystem for single-cell analysis.

2. Capsule networks are known to be computationally intensive due to dynamic routing. The manuscript does not report training time, GPU memory usage, or scalability with increasing cell or gene counts. It is suggested that the author add a runtime and memory comparison table or figure, and discuss scalability limitations and potential optimizations.

Answer: We thank the reviewer for this important and constructive suggestion. In response, we have added a new subsection in the revised version of the manuscript entitled **``Computational cost and scalability'**, and we have also added a detailed runtime and memory comparison in the Supplementary Material as Supplementary Table. Please see **page no 12** of the revised manuscript and **page no 2** of supplementary text.

In the revised analysis, we report the approximate training time and peak GPU memory usage of RareCapsNet and representative deep learning-based single-cell methods, including scVI, scANVI, CellAssign, and ItClust. All models were benchmarked on a PBMC68k-sized dataset using an NVIDIA RTX 3090 GPU with 24 GB memory. RareCapsNet required approximately 25 minutes of training time and 8.5 GB of peak GPU memory. This is moderately higher than scVI and scANVI, and also higher than CellAssign and ItClust in our benchmark, reflecting the additional cost of iterative routing between primary capsules and type capsules.

We have clarified in the revised manuscript that this overhead is primarily due to the dynamic routing layer and the fixed set of primary capsules. However, because the number of primary capsules and routing iterations are fixed across experiments, the model remains practical for datasets in the 50k--100k cell range after standard highly variable gene selection.

3. Cross-batch transfer learning setup is underspecified. It is unclear how gene space alignment is handled across datasets (e.g., CBMC → Pollen). If gene sets differ, direct input is infeasible. The authors should clarify:

Whether only intersecting genes were used; Whether any feature alignment or transfer layer was employed; If not, this should be noted as a limitation.

Answer:

We thank the reviewer for this important observation. In the revised manuscript, we have clarified the cross-batch transfer protocol in the subsection **``Rare cell identification in different batch of data''** and in the Supplementary Material under section **``Cross-Dataset**

Generalization'. Please see the page no 8-10 of the main manuscript and page no 3 of the supplementary text

For all cross-dataset transfer experiments, we restricted the input feature space to the intersection of genes shared between the source and target datasets. Specifically, each dataset was first processed using the same normalization and log-transformation pipeline. Highly variable genes were then identified, and the final transfer input was constructed using the common intersecting gene set between the source and target datasets. The intersected gene expression matrices were ordered identically before being provided to RareCapsNet. Therefore, the model was evaluated only on a matched gene space.

No additional feature-alignment module, transfer layer, or domain-adaptation layer was used. RareCapsNet was trained on the source dataset and then applied directly to the target dataset using the intersected gene space. We have now explicitly stated this in the main manuscript and noted it as a limitation. In particular, we now clarify that the current transfer setting is intended to evaluate whether capsule activations remain informative under a shared-gene representation, rather than to solve the more general problem of transfer across datasets with non-overlapping gene panels. We also added this point as a future direction, where batch-aware feature alignment, domain adaptation, or gene-embedding-based transfer layers could further improve cross-platform generalization.

4. I didn't see the labels for sub-figures A and B in Figure 2, but they are present in the figure caption.

Answer: We thank the reviewer for pointing this out. Since Figure 2 is a single composite figure rather than two explicitly labeled subfigures, we have revised the caption to avoid references to missing subfigure labels A and B. The updated caption now describes the upper block corresponding to simulation setting S1 and the lower block corresponding to simulation setting S2, with additional explanation of the t-SNE plots, coupling heatmaps, and interpretation of high coupling values.

5. Interpretability claims are insufficiently validated. While known markers (e.g., CLEC4C, LILRA4) are recovered, no validation is provided for newly suggested markers. The authors should consider: Overlap analysis with public marker databases. Gene set enrichment analysis (GO/KEGG) for capsule-associated gene lists. Literature evidence for top-ranked novel genes.

Answer:

We thank the reviewer for this important and constructive suggestion. In response, we have substantially expanded the section **“Marker-gene analysis”** in the revised manuscript.

First, we performed an overlap analysis between RareCapsNet-derived capsule-associated genes and public marker resources, including CellMarker, PanglaoDB, and curated PBMC/brain marker annotations. Second, we performed functional enrichment analysis of capsule-associated gene lists using GO Biological Process and KEGG pathway annotations. Third, we reviewed the top-ranked genes that were not included in the canonical marker lists and now describe them more conservatively as putative capsule-associated markers rather than definitive novel biomarkers.

Importantly, we have now moved the key validation results into the main manuscript under the subsection **``Marker gene analysis'**. **Table-6** summarizes representative capsule-associated genes, their overlap with public marker resources, and enriched biological functions. The extended validation details are provided in the Supplementary Material under the subsection **``Biological validation of capsule-associated marker genes'**. Please see **page no 11 of the main manuscript and page no. 3 of the supplementary text.**

The added analysis shows that capsule-associated genes are biologically coherent. For example, pDC-associated capsules recovered \textit{CLEC4C}, \textit{LILRA4}, \textit{TCF4}, and \textit{IRF7}, with enrichment for type I interferon signaling and antiviral response. Dendritic-cell-associated capsules recovered \textit{FCER1A}, \textit{CST3}, and \textit{HLA-DRA}, with enrichment for antigen presentation and leukocyte activation. Megakaryocyte-associated capsules recovered \textit{PF4}, \textit{PPBP}, \textit{ITGA2B}, and \textit{GP9}, with enrichment for platelet activation and coagulation. These additions strengthen the interpretability claim by demonstrating that RareCapsNet-derived gene-level explanations are consistent with known marker resources and relevant cellular functions.

Minor

1. It is recommended not to specifically emphasize "In-silico analysis of single cell data" as a downstream analysis in the abstract.

Answer: We thank the reviewer for this suggestion. We agree that the opening sentence of the abstract was too general and unnecessarily emphasized the term ``in-silico analysis'' as downstream analysis. We have revised the abstract to begin directly with the biological and computational challenge addressed by the manuscript, namely rare cell identification in large-scale single-cell RNA-seq data.

2. The "figure" in the manuscript should be changed to "Figure".

Answer: As advised we have updated the word “figure”to the word “Figure” in the revised version of the manuscript.

3. Figure 2 and 3: Heatmap and UMAP color scales are not clearly labeled.

Answer: We thank the reviewer for pointing this out. We have now revised Figures 2 and 3 to improve readability of the heatmaps and UMAP plots. We have also expanded the corresponding figure captions to explain how the color scale should be interpreted.

4. Several results are “shown in supplementary” but no supplementary file is currently provided in the manuscript bundle.

We apologize for this omission. We have now included the complete Supplementary Material file with the revised manuscript bundle.

Answer to the Reviewer-2

The manuscript proposes RareCapsNet, an explainable capsule network framework for the identification of rare cell populations from large-scale single-cell RNA sequencing data. The study addresses an important and challenging problem in single-cell transcriptomics, namely the reliable detection of rare cellular subpopulations under conditions of extreme class imbalance and high dropout.

Overall, the manuscript presents a novel methodological contribution, and the proposed framework shows promising results across simulated and real datasets.

Despite these strengths, several aspects of the manuscript would benefit from further clarification and refinement before publication.

1. The description of the model architecture could be expanded to improve reproducibility. While the main components of the capsule network are described, additional details regarding hyperparameter selection, routing iterations, and architectural design choices would help readers better understand how the model was implemented and optimized. A short ablation study examining the influence of key parameters (such as number of capsules or routing depth) would strengthen the methodological section.

Answer: We thank the reviewer for this constructive suggestion. In response, we have expanded the methodological description of RareCapsNet to include the main architectural components, the number of primary capsules, the number of type capsules, routing iterations, training configuration, and the rationale behind the final design choices. Please see the updated subsection ``**Architecture and training objective of RareCapsNet**" and newly added subsections ``**Definition of primary capsules and type capsules.**" and within the Method section (see page no 12-13).

In addition, we have added an ablation analysis evaluating the influence of two key capsule-network parameters: the number of primary capsules and the number of dynamic routing iterations. The main ablation results are now included in the revised main manuscript in the subsection ``**Ablation analysis of capsule-network parameters'** (page no 7 and Table-3). A more detailed explanation is provided in the supplementary materials (section ``Ablation Study – Effect of Capsule Parameters", page no 4-5).

The ablation results show that performance improves as the number of primary capsules increases from 8 to 32, with the highest F1-score obtained at 32 primary capsules. Increasing the

number of capsules to 64 does not further improve performance, suggesting that excessive capsule capacity may reduce specialization. Similarly, performance improves from 1 to 3 routing iterations and then saturates, indicating that 3 routing iterations are sufficient for stable capsule assignment. These results support the architectural configuration used throughout the manuscript: 32 primary capsules and 3 routing iterations.

2. The evaluation currently compares RareCapsNet with several established rare-cell detection approaches such as FiRE, GiniClust, RaceID, and CellSIUS. While these comparisons are appropriate, the manuscript may benefit from additional comparisons with recent deep learning–based representation learning methods for single-cell data, such as autoencoder-based frameworks or graph neural network approaches.

Answer: We thank the reviewer for this valuable suggestion. In response, we have added a new subsection in the Results section entitled ``**Comparison with deep learning-based single-cell methods'' (page no 10)**. In this subsection, we benchmark RareCapsNet against four widely used deep learning-based methods: **scVI, scANVI, CellAssign,** and **ItClust**, in addition to the classical rare-cell detection methods already considered. The additional details retained in the Supplementary Material (subsection: **"Additional details on deep learning-based baseline comparisons", page no.1**).

These methods were selected because they represent widely used and complementary deep learning strategies for single-cell analysis. scVI is a variational autoencoder-based generative model for single-cell transcriptomics, scANVI extends this framework to semi-supervised annotation and harmonization, CellAssign is a probabilistic marker-informed cell-type assignment method, and ItClust is a neural network-based transfer learning method for clustering and cell-type classification. We evaluated these methods on PBMC68k, CBMC, Zeisel mouse brain, and a cross-batch transfer setting, using F1-score for rare-cell detection as the main evaluation metric.

We believe that this additional comparison strengthens the manuscript by positioning RareCapsNet within the broader landscape of modern deep learning methods for single-cell analysis, while also highlighting its specific advantage for interpretable rare-cell identification.

3. The manuscript briefly discusses cross-dataset transferability of the learned capsule representations. This is an interesting property of the model, but the discussion could be expanded to provide more quantitative analysis and interpretation.

Answer:

We thank the reviewer for this helpful suggestion. In the revised manuscript, we have expanded the subsection **``Rare cell identification in different batches of data''** (page no 8-10) to provide a more explicit description of the transfer protocol, gene-space matching, and quantitative performance. Additional details are also retained in the subsection**``Cross-Dataset Generalization”** in the supplementary text. (page no 3)

Specifically, we now clarify that for each source--target transfer experiment, datasets were first processed using a consistent normalization and log-transformation pipeline, and the input gene space was restricted to the common intersecting genes shared between the source and target datasets. The intersected gene expression matrices were ordered identically before applying the trained RareCapsNet model to the target dataset. No additional feature-alignment layer, domain-adaptation module, or fine-tuning was used.

We also added a quantitative summary table in the main text showing representative transfer results, including source dataset, target dataset, feature-alignment strategy, retraining status, and transfer performance. Under this setting, RareCapsNet retained strong rare-cell detection performance, including $>85\%$ accuracy for Darmanis-to-Pollen transfer and 91\% accuracy for CBMC-to-Yan transfer. We have also expanded the interpretation to emphasize that the current transfer analysis supports the stability of learned capsule activations under a shared-gene representation, while also noting that transfer across substantially non-overlapping gene panels remains a limitation and future direction.

4. Since large-scale single-cell datasets often contain hundreds of thousands of cells, a short discussion about the computational cost of RareCapsNet would be useful. Providing approximate training time, memory requirements, or scalability characteristics would help readers assess how the method can be applied to very large datasets.

Answer:

We thank the reviewer for this important suggestion. In response, we have added a new subsection in the main manuscript entitled **``Computational cost and scalability''** (page no 11), and we have included a detailed runtime and GPU memory comparison in the Supplementary Material as section **``Runtime, memory usage, and scalability”** (page no 2).

In the revised manuscript, we now report approximate training time and peak GPU memory usage for RareCapsNet and representative deep learning-based single-cell methods, including scVI, scANVI, CellAssign, and ItClust. All methods were benchmarked on a PBMC68k-sized dataset using an NVIDIA RTX 3090 GPU with 24 GB memory. RareCapsNet required approximately 25 minutes of training time and 8.5 GB of peak GPU memory. This reflects the

additional computational cost introduced by iterative dynamic routing between primary capsules and type capsules.

We have also clarified that the main training cost scales approximately linearly with the number of cells and input genes after highly variable gene selection, since the number of primary capsules and routing iterations is fixed across experiments. We further note that the post hoc gene-attribution step is more computationally demanding because it requires repeated masked forward passes, but this step is performed after model training and can be parallelized across genes.

Finally, we have added a brief discussion of scalability limitations and possible optimization strategies, including sparse routing, shared transformation matrices, mini-batch-level routing approximation, and GPU-parallelized gene attribution. We believe these additions make the computational requirements and scalability characteristics of RareCapsNet clearer for readers interested in applying the method to larger single-cell datasets.

5. The manuscript briefly discusses transferability of the model across different datasets. This is an interesting and potentially impactful property of RareCapsNet. The authors may consider expanding this section slightly to provide additional details about: how datasets were aligned or normalized before transfer, whether batch effects influence capsule activation patterns.

Answer:

We thank the reviewer for this insightful suggestion. We agree that the transferability analysis required a clearer description of dataset normalization, gene-space alignment, and the possible influence of batch effects on capsule activation patterns. In response, we have expanded the subsection **``Rare cell identification in different batches of data'' (page no 8-10)** in the main manuscript and added further details in the Supplementary Material under **``Cross-Dataset Generalization''**.

In the revised manuscript, we now clarify that all datasets used in the transfer experiments were processed using a consistent preprocessing pipeline, including library-size normalization, log-transformation, and highly variable gene selection. Since source and target datasets may contain different gene sets, we restricted each transfer experiment to the common intersecting genes shared by the two datasets. The intersected gene matrices were ordered identically before applying the trained RareCapsNet model to the target dataset. Thus, transfer was performed only in a matched gene space.

We also clarified that no explicit batch-correction method, domain-adaptation layer, feature-alignment layer, or fine-tuning step was used. The purpose of the analysis was to

evaluate whether capsule activations learned from one dataset remain informative when applied to another dataset represented using the shared gene space. We observed that although batch effects may slightly alter activation magnitudes, the relative capsule activation patterns associated with rare or underrepresented populations remained stable across source and target datasets. This suggests that RareCapsNet captures biologically meaningful capsule-level representations that can be partially transferred across related datasets.

At the same time, we have explicitly noted this as a limitation. The current implementation assumes the availability of a sufficiently large shared gene set and does not yet address transfer across datasets with largely non-overlapping gene panels or strong platform-specific effects. We now discuss batch-aware capsule routing, domain adaptation, and gene-embedding-based alignment as possible future extensions to improve cross-dataset transferability.

6. In some sections, terms such as cell types, cell classes, and type capsules are used interchangeably. Maintaining consistent terminology throughout the manuscript would improve clarity.

Answer:

We thank the reviewer for pointing out this important issue. We agree that the previous use of ``cell types'', ``cell classes'', and ``type capsules'' could cause ambiguity between biological annotations and model components. In the revised manuscript, we have carefully standardized the terminology throughout the main text, Methods, figure captions, and Supplementary Material.

Specifically, we now use \textit{cell type} to refer to biological cell identities or annotations, such as pDCs, dendritic cells, monocytes, or megakaryocytes. We use \textit{type capsule} only to refer to the output capsule in the RareCapsNet architecture corresponding to a predicted cell type. The term \textit{cell class} has been avoided except in simulation-specific contexts where it refers to an artificial label category, such as two-class or three-class simulated datasets.

We have also added clarifying language in the Methods section to explicitly define primary capsules and type capsules. This revision improves conceptual clarity by distinguishing biological entities from model components.

7. Some figure captions could be expanded to improve interpretability. In particular, figures showing capsule activations or coupling coefficients would benefit from more detailed descriptions explaining how readers should interpret the visual pattern.

Answer:We thank the reviewer for this helpful suggestion. We agree that figures involving capsule activations and coupling coefficients require more explicit captions so that readers can understand how to interpret the visual patterns. In the revised manuscript, we have expanded the

captions of the relevant figures, particularly those showing t-SNE/UMAP plots, coupling heatmaps, and capsule activation patterns.

Specifically, the revised captions now explain that coupling heatmaps represent the routing strength between primary capsules and type capsules, where darker or more intense colors indicate stronger coupling. We also clarify that rows or axes corresponding to primary capsules should be interpreted as learned capsule-level representations, while type capsules correspond to predicted cell types. For the simulation figures, we now describe how increasing rare-cell imbalance affects capsule specialization, and for the real-data figures we explain how strong activation or high coupling values indicate capsule association with rare cell populations.

8. Although the datasets used in the experiments are well known, it would be helpful to include a short table summarizing key properties of each dataset (number of cells, number of genes, number of cell types, proportion of rare populations).

Answer: We thank the reviewer for this useful suggestion. We agree that a concise dataset summary table improves clarity and helps readers understand the scale and rarity structure of each benchmark. In response, we have added a new table in the Supplementary Material, **Supplementary Table-6 (page no 7)**, summarizing the key properties of all real datasets used in the study. The table includes the number of cells, number of genes/features used after preprocessing, number of annotated cell types, rare populations considered, and their approximate proportions. We also added a short explanatory paragraph in the Supplementary Material, section ``**Public scRNA-seq Datasets" (page no 5)** to clarify that the gene counts refer to the processed feature space used for model training, typically after highly variable gene selection.

9. The discussion section could be slightly expanded to emphasize broader implications of the framework and potential future extensions.

Answer: We appreciate the reviewer's suggestion. Accordingly, we have revised and expanded the **Conclusion** section to articulate broader implications of RareCapsNet. We outline concrete future directions, including (1) semi-supervised learning, (2) multimodal extensions to integrate CITE-seq or spatial data, (3) model compression for scalable applications, and (4) unsupervised capsule routing for rare cell discovery without labels. These extensions build on RareCapsNet's core strengths in interpretability and robustness, and demonstrate its potential to contribute to atlas-level and clinical-scale single-cell analysis workflows.

10. The manuscript mentions the availability of the implementation. Providing more

details regarding dependencies, runtime environment, and example scripts would further facilitate adoption by the research community.

Answer: We thank the reviewer for this helpful suggestion. In response, we have updated the supplementary material and the GitHub repository (linked in the manuscript) to include the following:

- A README.md file with detailed instructions on installation, runtime dependencies (Python 3.8+, PyTorch ≥1.10, NumPy, Scanpy, Linnorm for preprocessing), and tested OS environments (Ubuntu 20.04, Windows 11).
- Step-by-step example scripts for running RareCapsNet on both simulated and real datasets, including preprocessing, model training, and interpretability modules.
- Environment setup via conda and requirements.txt for quick deployment.

Answer to the Reviewer-3

This manuscript proposed RareCapsNet, a method based on capsule networks for discovering rare cell populations with an emphasis on explainability. It is compared with GiniClust, FiRE, RaceID, and CellSIUS, using both simulated datasets and several real scRNA-seq datasets, such as Jurkat, CBMC, PBMC, and Mouse brain dataset. The topic is interesting and potentially important for rare cell type detecting in single-cell analysis. However, the manuscript requires substantial improvements in clarity, methodological description, and presentation before it can be considered further. My detailed comments are listed below:

Major concerns:

1. After carefully reading the manuscript, the overall framework of RareCapsNet is still difficult to understand. The procedures presented in Figure 1 are not sufficiently clear. The training objective (such as loss function) is not explicitly described in the text. I recommend that the authors could include a section describing the complete workflow in detail. In particular, the mathematical formulation of each layer (with its size) and the training procedure should be clearly defined.

Answer: We thank the reviewer for this important and constructive comment. We agree that although the manuscript already contained a subsection entitled **``Workflow of RareCapsNet''** in the Results section, the previous version did not provide sufficient mathematical and implementation-level details of the model architecture and training objective.

To address this without duplicating the workflow description, we retained the existing **``Workflow of RareCapsNet"** subsection as a conceptual overview linked to Figure~1, and added a new subsection in the Method section entitled **``Architecture and training objective of RareCapsNet'' (page no 12-13)**. This new subsection provides a formal layer-wise description of the model, including the input expression vector, fully connected projection layer, primary capsule layer, type capsule layer, dynamic routing equations, prediction rule, and training loss.

We now explicitly define the dimensions of the main model components: the input gene vector of dimension K, the fixed number of primary capsules $J=32$, the number of type capsules T corresponding to the number of annotated cell types, and the use of three routing iterations. We also added the margin-based capsule loss used for supervised training and clarified the training protocol, including train/validation/test splitting, mini-batch optimization, and validation-based model selection. These additions make the implementation and optimization of RareCapsNet more reproducible and clarify how Figure~1 relates to the mathematical model.

2. Is RareCapsNet a supervised learning method? If the model relies on labeled data, its applicability for detecting rare cell types in new datasets may be limited. But the authors claimed that the method can identify "novel rare cell population" in the abstract. The authors should also clarify how to distinguish novel cell types with outliers.

Answer: We thank the reviewer for raising this important conceptual point. We agree that the supervised nature and applicability of RareCapsNet should be stated more clearly. RareCapsNet, in its current implementation, is primarily a supervised learning framework. It requires labeled training data to learn the association between gene-expression patterns, primary capsules, and type capsules. Therefore, its strongest and most direct use case is the robust detection of rare, poorly covered, or underrepresented cell types that are present in the training annotations or in a related reference dataset.

In response, We have added this clarification in the Method section under the paragraph **``Supervised setting and candidate outlier detection''** (page no 13) and have also revised the abstract and Conclusions to avoid overclaiming fully de novo rare-cell discovery. Specifically, we replaced phrases such as ``novel rare cell population'' with more precise wording such as ``putative rare cell populations'', ``poorly represented rare cell populations'', or ``rare cell populations represented in the training/reference labels''. We now explicitly state that RareCapsNet should not be interpreted as a fully unsupervised novel cell-type discovery method in its current form.

To clarify how potentially novel populations or outliers could be handled, we have added a short paragraph in the Methods/Discussion explaining that cells with low maximum type-capsule activation, diffuse coupling patterns, or weak assignment to all known type capsules may be flagged as outlier or candidate novel cells for downstream validation. However, such cells require additional post hoc analysis, including clustering, marker-gene inspection, and biological validation, before being interpreted as novel cell types. Thus, RareCapsNet can support the identification of candidate outlier or putative novel populations, but definitive novel cell-type discovery remains a future extension requiring semi-supervised or open-set learning.

3. I'm still confused with the concepts of "primary capsule", "type capsule". Does the number of primary capsules correspond to feature groups or feature types? These terms should be clearly defined with in the methodological workflow (a detailed workflow as comment 1).

We thank the reviewer for pointing out this source of confusion. In the revised Method section, particularly in the new subsection **``Architecture and training objective of RareCapsNet''** (**page no 12-13**), we have added explicit definitions of both capsule types.

In RareCapsNet, **primary capsules** are intermediate learned vector representations produced after the fully connected projection layer. They do not correspond to pre-defined biological feature groups, gene modules, or manually specified marker categories. Rather, they are trainable latent capsule units that learn gene-expression patterns from the data. The number of primary capsules is therefore a model hyperparameter. In the final RareCapsNet architecture, we use 32 primary capsules..

By contrast, **type capsules** are the output capsules of the model. The number of type capsules is equal to the number of annotated biological cell types in the training dataset. Each type capsule represents one candidate cell type, and the predicted cell identity is assigned based on the type capsule with the largest activation norm.

We have also clarified that coupling coefficients quantify the routing strength from each primary capsule to each type capsule. Thus, high coupling between a primary capsule and a type capsule indicates that the learned expression pattern represented by that primary capsule contributes strongly to the prediction of the corresponding cell type. We believe these additions make the model workflow and terminology clearer.

4. The "dynamic routing procedure" is not clearly described? Does Equation (2) represent its formal definition? A clearer explanation would be helpful.

Answer: We thank the reviewer for this important clarification. In the revised manuscript, we have expanded the Method section under **``Architecture and training objective of RareCapsNet'' (page no 12-13)** to provide a complete mathematical description of dynamic routing. We now explicitly define: (i) prediction vectors from primary capsules to type capsules, (ii) routing logits, (iii) softmax-normalized coupling coefficients, (iv) type capsule input vectors, (v) the squash nonlinearity, and (vi) the iterative agreement-based update of routing logits. We also clarify that dynamic routing is an iterative procedure repeated for a fixed number of routing iterations, which was set to three based on the ablation analysis.

We now state that the previous Equation (2) corresponds only to the coupling-coefficient normalization step within dynamic routing, not the entire routing algorithm. The full dynamic routing procedure is now described by the set of equations defining prediction vectors, coupling coefficients, type capsule outputs, and routing-logit updates.

5. The manuscript claims explainability, but the definition is unclear. Is the explainability measured by the average correlation between primary capsules and cell-type capsules (as illustrated in Figure 1(D) and Eq.(1) in the Methods section)? Additionally, does the term 1/N(t) aim to compensate for differences in cell counts between cell types?

Answer: We thank the reviewer for this important clarification. As advised by the reviewer, in the revised manuscript, we clarify that RareCapsNet explainability is not measured by a correlation coefficient. Rather, it is based on the dynamic-routing coupling coefficients between primary capsules and type capsules. These coupling coefficients quantify how strongly a learned primary capsule contributes to a given type capsule during prediction.

Specifically, after training, we identify primary capsules that show strong coupling to a given type capsule. We then compute gene-specific coupling coefficients by masking all genes except one and measuring how the coupling between a selected primary capsule and type capsule changes. In this way, genes that produce high coupling values are interpreted as contributing strongly to the capsule-level representation of that cell type. Thus, the explainability mechanism consists of two linked steps: (i) identifying cell-type-associated primary capsules using routing coefficients, and (ii) attributing genes to those capsules using gene-specific masked-input coupling scores.

We also thank the reviewer for pointing out the role of the term $1/N(t)$ in Eq.~(1) (now Eq. 12 in the revised version). Yes, the term $1/N(t)$ is included to average coupling values within each cell type and to prevent abundant cell types from dominating the gene-specific coupling score. The subsequent factor $1/T$ gives equal weight to each cell type. Therefore, Eq.~(1) (now Eq. 12 in the revised version) performs stratified averaging across cell types, which is particularly important in rare-cell settings where cell-type frequencies are highly imbalanced. We have revised the Methods section to explicitly state this point. Please see the paragraphs **"Gene-Specific Coupling Coefficient"** and paragraph **"Coupling-based definition of explainability."** under the subsection **"Theoretical Foundations of RareCapsNet Gene Attribution" (page no 14)** in the revised version of the manuscript.

6. The figures require significant improvement.
(1) The text within the figures is too small and difficult to read. Important elements should be enlarged or highlighted.
(2) Some symbols are missing or unclear, e.g., the notation "N" in Figure 1(A) appears incomplete. Does "N" represent the number of cells with one feature to train? Please clarify the details. In Figure 1(B), it is unclear whether the input corresponds to a single cell or multiple cells. (As comment 1, a detailed workflow is necessary)

Answer: We thank the reviewer for these important comments. We have now revised the figures to enlarge the text, increase label size, and improve the visibility of key elements. Important modules and flow arrows have been highlighted more clearly so that the figure can be understood without ambiguity.

We have also clarified the notation used in Figure~1. In Figure~1(A), N denotes the number of cells (samples) in the dataset, while K denotes the number of genes/features used as input after preprocessing. Thus, the input expression matrix is of size $N \times K$, where rows correspond

to cells and columns correspond to genes. We have corrected the figure annotation so that this is now explicitly stated in the revised figure.

In addition, we clarified Figure~1(B) to indicate that the capsule network operates on \emph{one single-cell expression vector at a time} during model training and inference. That is, the input to the network is a single cell represented by a K-dimensional gene-expression vector, while the full dataset of N cells is used across training batches. We have revised the figure labels and the corresponding text/caption to make this distinction explicit.

To further address the reviewer's concern, we also expanded the caption of Figure~1 and added a more detailed methodological subsection in the revised manuscript describing the full workflow, layer-wise architecture, and training procedure. We believe these changes substantially improve both figure readability and conceptual clarity.

7. (1) In Data S2 and Data S4, the dropout rate is ~0.5 (approximately or exactly?) in the text. But it is reported as 0.4 in Table 1 and in SI. This inconsistency should be clarified.
(2) For Data S5, it is unclear whether "four samples" (used in the text) or "four classes" (used in the table) are simulated. The statement "lower than 10%" is inaccurate (for example 0.01 is also <10%).

Answer: We thank the reviewer for carefully pointing out these inconsistencies. We have corrected the simulation description, Table 1, and the Supplementary Information accordingly.

Second, for S5, the phrase ``four samples'' was incorrect. We have replaced it with ``four cell types/classes''. We have also revised the vague phrase ``lower than 10\%'' by explicitly specifying the proportions of the four rare cell types. In the revised version, S5 contains 13 simulated cell types, of which four are rare with proportions 0.5\%, 1\%, 2\%, and 5\%, while the remaining nine cell types share the remaining cells approximately equally. This clarification removes ambiguity and makes the rarity structure of S5 reproducible.

8. The result shown in Figure 3(A) is confusing. Why does Leiden clustering fail to cluster in this case. (One cell belonging to Cluster-2 appears in Cluster 1, and three cells belonging to Cluster-1 are surrounded by Cluster-2.) More explanation is needed.

Answer: We thank the reviewer for pointing out this ambiguity. In the revised manuscript, we have clarified that Leiden clustering largely separates the two dominant transcriptomic groups in the Jurkat--293T mixture, but it produces a small number of locally ambiguous or misassigned cells under the extreme class-imbalance setting.

We have revised the explanation of Figure~3(A) to clarify two points. First, Leiden clustering is performed on a neighborhood graph, whereas the UMAP is a two-dimensional visualization of that graph structure; therefore, apparent visual proximity in UMAP does not always correspond exactly to the graph connectivity used by Leiden. Second, because the Jurkat population represents only a small fraction of the total cells, a few cells with intermediate or noisy expression profiles may be connected to the majority-cell neighborhood and assigned to the opposite cluster. This explains the few visually discordant points.

We have revised the figure caption and the corresponding Results text to state more precisely that Leiden clustering shows minor cluster contamination under rarity and expression-noise effects, whereas RareCapsNet is evaluated quantitatively for rare-cell detection using F1-score and recall in Figure~3(C). This revision should make the interpretation of Figure~3(A) clearer.

9. Page 7: "RareCapsNet successfully identified rare subpopulations". How to define "successful"? The criteria for defining successful identification should be clearly stated, especially for rare cell populations.
Page 7: "biologically meaningful gene sets". The authors should do the functional enrichment analysis to support this claim.

Answer: We thank the reviewer for this important clarification. By ``successful identification", we mean that a rare population is detected with strong quantitative performance on held-out cells, specifically high rare-class F1-score together with consistent precision and recall, and that the prediction is supported by distinct primary capsule activation patterns and biologically interpretable capsule-associated genes.

To avoid any ambiguity, we have revised the manuscript to either remove the phrase ``successfully identified" or replace it with evidence-based wording. Specifically, rare-cell detection is now described in terms of rare-class F1-score, precision/recall, distinct capsule activation patterns, and biological validation of capsule-associated genes.

For the statement regarding ``biologically meaningful gene sets", we have added marker-resource overlap and GO/KEGG functional enrichment analysis. The revised manuscript now reports that capsule-associated genes show enrichment for biologically relevant functions, including type I interferon signaling for pDCs, antigen processing and presentation for dendritic cells, platelet activation for megakaryocytes, and GABAergic synaptic signaling or neuronal differentiation for neurogliaform/interneuron-like cells. These results are summarized in **Table-6**, with additional details provided in the Supplementary Material. We believe these revisions clarify the basis of rare-cell identification and support the biological interpretation of the capsule-associated genes.

10. Page 7: (1) "These results confirm that RareCapsNet is suitable for cross-dataset applications without the need for full retraining."
The evidence (e.g. 85% or 91% accuracy for the whole dataset?) provided is not sufficient

to support such a strong conclusion. The authors should present the results more objectively.

Answer: We thank the reviewer for this important comment. We agree that the previous wording was too strong. In particular, the phrase ``confirm that RareCapsNet is suitable for cross-dataset applications without the need for full retraining'' could overstate the generality of the transfer result. In the revised manuscript, we have softened this statement and now present the cross-dataset analysis more objectively.

We have clarified that the reported 85\% and 91\% values refer to transfer performance in the evaluated source--target dataset pairs under a matched-gene setting, rather than a universal guarantee of cross-dataset performance across all datasets or platforms. We have also revised the text to state that these results \textit{suggest} that capsule activations can retain useful rare-cell information across related datasets represented in a shared gene space.

In addition, we explicitly note the limitations of the current transfer experiment. The analysis assumes the availability of a sufficiently large set of intersecting genes between source and target datasets, and no dedicated domain-adaptation or gene-space alignment layer was used. Therefore, cross-dataset transfer across substantially different platforms or non-overlapping gene panels remains an important future direction. We have revised the relevant Results and Discussion text accordingly.

(2) "Yan19, Pollen20, Darmanis21, and CBMC22 (see supplementary text for the detailed description of the datasets)". I searched the word "Yan/Pollen..." in the SI, but could not find the corresponding descriptions in the SI. Please make a clear statement or citation.

Answer: We thank the reviewer for carefully checking this. We apologize for the omission. In the revised Supplementary Information, we have added a dedicated subsection entitled **``Datasets used for cross-dataset transfer analysis''**, where we now provide short descriptions and citations for the Yan, Pollen, Darmanis, and CBMC datasets. We have also revised the corresponding sentence in the main manuscript to make the reference to the Supplementary Information clearer. The main text now states that detailed descriptions of these datasets are provided in the Supplementary Information under the subsection \textit{``Datasets used for cross-dataset transfer analysis''}.

(3) I supposed that CBMC used as the training dataset was labeled and obtained from the same tissue as the other three datasets. If the number of cell types differs across datasets, can the model detect new rare cell types that were not present during training?

Answer: We thank the reviewer for raising this important conceptual point. RareCapsNet, in its current form, is a supervised model: the number of type capsules is determined by the number of cell types present in the labeled training dataset. Therefore, if a target dataset contains a cell type

that was not represented during training, RareCapsNet cannot directly assign that cell to a new type capsule, because no output capsule corresponding to that unseen cell type exists.

We have revised the manuscript to clarify this limitation. The cross-dataset transfer analysis is intended to evaluate whether capsule representations learned from a source dataset remain informative when applied to a target dataset represented in a shared gene space. It should not be interpreted as a fully open-set or de novo cell-type discovery framework. When source and target datasets differ in cell-type composition, direct classification is meaningful only for shared or harmonized cell-type labels. Cells belonging to target-only cell types may be assigned to the closest known type capsule, or may show low maximum capsule activation and diffuse coupling patterns. Such cells can be flagged as candidate outliers or putative novel populations, but they require additional downstream validation, including clustering, marker-gene inspection, and comparison with reference marker databases.

We have now explicitly stated that detection of genuinely unseen rare cell types requires future extensions such as open-set recognition, uncertainty-aware rejection thresholds, semi-supervised learning, or the addition of new type capsules through fine-tuning. We have revised the cross-dataset transfer section accordingly to avoid overclaiming the ability to identify cell types absent from the training labels.
