# Supplementary material for: RareCapsNet: An explainable capsule network enables robust discovery of rare cell populations from large-scale single-cell transcriptomics
Source: PLoS Comput Biol. 2026 Aug 10;22(8):e1013962. doi: 10.1371/journal.pcbi.1013962 (PMC13472507; doi:10.1371/journal.pcbi.1013962)
Supplement: Supplementary file 3 [file pcbi.1013962.s002.docx]

**General**

This This manuscript introduces RareCapsNet, a capsule network-based framework for the identification of rare cell populations from single-cell RNA-seq data. The model leverages dynamic routing to model part-whole relationships between genes, primary capsules, and cell-type capsules. However, several methodological inconsistencies, missing baselines, and reproducibility concerns need to be addressed before the manuscript can be accepted for publication.

**Major**

1. The comparison is limited to classical rare-cell tools (RaceID, GiniClust, FiRE). No deep learning-based baselines (e.g., scVI, scANVI, CellAssign, ItClust) are included. This is particularly concerning for the PBMC68k and cross-batch experiments, where deep generative models are now standard. It is recommended to add at least 2–3 modern DL-based methods to the benchmark.
2. Capsule networks are known to be computationally intensive due to dynamic routing. The manuscript does not report training time, GPU memory usage, or scalability with increasing cell or gene counts. It is suggested that the author add a runtime and memory comparison table or figure, and discuss scalability limitations and potential optimizations.
3. Cross-batch transfer learning setup is underspecified. It is unclear how gene space alignment is handled across datasets (e.g., CBMC → Pollen). If gene sets differ, direct input is infeasible. The authors should clarify:

Whether only intersecting genes were used;

Whether any feature alignment or transfer layer was employed;

If not, this should be noted as a limitation.

4. I didn't see the labels for sub-figures A and B in Figure 2, but they are present in the figure caption.
5. Interpretability claims are insufficiently validated. While known markers (e.g., CLEC4C, LILRA4) are recovered, no validation is provided for newly suggested markers. The authors should consider:

Overlap analysis with public marker databases.

Gene set enrichment analysis (GO/KEGG) for capsule-associated gene lists.

Literature evidence for top-ranked novel genes.

**Minor**

1. It is recommended not to specifically emphasize "In-silico analysis of single cell data" as a downstream analysis in the abstract.
2. The "figure" in the manuscript should be changed to "Figure".
3. Figure 2 and 3: Heatmap and UMAP color scales are not clearly labeled.
4. Several results are “shown in supplementary” but no supplementary file is currently provided in the manuscript bundle.
