# Supplementary material for: RareCapsNet: An explainable capsule network enables robust discovery of rare cell populations from large-scale single-cell transcriptomics
Source: PLoS Comput Biol. 2026 Aug 10;22(8):e1013962. doi: 10.1371/journal.pcbi.1013962 (PMC13472507; doi:10.1371/journal.pcbi.1013962)
Supplement: Peer review history

# Peer review: Decision Letter 0

**DOI:** 10.1371/journal.pcbi.1013962.r001

**Authors:** Pedro Mendes, Xiaoqiang Sun

17 Mar 2026

PCOMPBIOL-D-26-00254

RareCapsNet: An explainable capsule networks enable robust discovery of rare cell populations from large-scale single-cell transcriptomics

PLOS Computational Biology

Dear Dr. Ray,

Thank you for submitting your manuscript to PLOS Computational Biology. After careful consideration, we feel that it has merit but does not fully meet PLOS Computational Biology's publication criteria as it currently stands. Therefore, we invite you to submit a revised version of the manuscript that addresses the points raised during the review process.

Please submit your revised manuscript by May 17 2026 11:59PM. If you will need more time than this to complete your revisions, please reply to this message or contact the journal office at ploscompbiol@plos.org. When you're ready to submit your revision, log on to https://www.editorialmanager.com/pcompbiol/ and select the 'Submissions Needing Revision' folder to locate your manuscript file.

Please include the following items when submitting your revised manuscript:

* A letter that responds to each point raised by the editor and reviewer(s). You should upload this letter as a separate file labeled 'Response to Reviewers'. This file does not need to include responses to formatting updates and technical items listed in the 'Journal Requirements' section below.

* A marked-up copy of your manuscript that highlights changes made to the original version. You should upload this as a separate file labeled 'Revised Manuscript with Track Changes'.

* An unmarked version of your revised paper without tracked changes. You should upload this as a separate file labeled 'Manuscript'.

If you would like to make changes to your financial disclosure, competing interests statement, or data availability statement, please make these updates within the submission form at the time of resubmission. Guidelines for resubmitting your figure files are available below the reviewer comments at the end of this letter

We look forward to receiving your revised manuscript.

Kind regards,

Xiaoqiang Sun

Academic Editor

PLOS Computational Biology

Pedro Mendes

Section Editor

PLOS Computational Biology

**Additional Editor Comments:**

Please ensure that all the reviewers' comments are addressed and their questions are fully answered.

**Journal Requirements:**

1) Please ensure that the CRediT author contributions listed for every co-author are completed accurately and in full.

At this stage, the following Authors/Authors require contributions: Sumanta Ray, and Snehalika Lall. Please ensure that the full contributions of each author are acknowledged in the "Add/Edit/Remove Authors" section of our submission form.

The list of CRediT author contributions may be found here: https://journals.plos.org/ploscompbiol/s/authorship#loc-author-contributions

2) We ask that a manuscript source file is provided at Revision. Please upload your manuscript file as a .doc, .docx, .rtf or .tex. If you are providing a .tex file, please upload it under the item type u2018LaTeX Source Fileu2019 and leave your .pdf version as the item type u2018Manuscriptu2019.

3) Please provide an Author Summary. This should appear in your manuscript between the Abstract (if applicable) and the Introduction, and should be 150-200 words long. The aim should be to make your findings accessible to a wide audience that includes both scientists and non-scientists. Sample summaries can be found on our website under Submission Guidelines:

https://journals.plos.org/ploscompbiol/s/submission-guidelines#loc-parts-of-a-submission

4) Please upload all main figures as separate Figure files in .tif or .eps format. For more information about how to convert and format your figure files please see our guidelines:

https://journals.plos.org/ploscompbiol/s/figures

5) We have noticed that you have uploaded Supporting Information files, but you have not included a list of legends. Please add a full list of legends for your Supporting Information files after the references list.

6) We note that your Data Availability Statement is currently as follows: "provided in the github". Please confirm at this time whether or not your submission contains all raw data required to replicate the results of your study. Authors must share the “minimal data set” for their submission. PLOS defines the minimal data set to consist of the data required to replicate all study findings reported in the article, as well as related metadata and methods (https://journals.plos.org/plosone/s/data-availability#loc-minimal-data-set-definition).

For example, authors should submit the following data:

1) The values behind the means, standard deviations and other measures reported;

2) The values used to build graphs;

3) The points extracted from images for analysis..

Authors do not need to submit their entire data set if only a portion of the data was used in the reported study.

If your submission does not contain these data, please either upload them as Supporting Information files or deposit them to a stable, public repository and provide us with the relevant URLs, DOIs, or accession numbers. For a list of recommended repositories, please see https://journals.plos.org/plosone/s/recommended-repositories.

If there are ethical or legal restrictions on sharing a de-identified data set, please explain them in detail (e.g., data contain potentially sensitive information, data are owned by a third-party organization, etc.) and who has imposed them (e.g., an ethics committee). Please also provide contact information for a data access committee, ethics committee, or other institutional body to which data requests may be sent. If data are owned by a third party, please indicate how others may request data access.

**Reviewers' comments:**

Reviewer's Responses to Questions

**Comments to the Authors:**

**Please note here if the review is uploaded as an attachment.**

Reviewer #1: The review is uploaded as an attachment.

Reviewer #2: The manuscript proposes RareCapsNet, an explainable capsule network framework for the identification of rare cell populations from large-scale single-cell RNA sequencing data. The study addresses an important and challenging problem in single-cell transcriptomics, namely the reliable detection of rare cellular subpopulations under conditions of extreme class imbalance and high dropout.

Overall, the manuscript presents a novel methodological contribution, and the proposed framework shows promising results across simulated and real datasets.

Despite these strengths, several aspects of the manuscript would benefit from further clarification and refinement before publication.

1. The description of the model architecture could be expanded to improve reproducibility. While the main components of the capsule network are described, additional details regarding hyperparameter selection, routing iterations, and architectural design choices would help readers better understand how the model was implemented and optimized. A short ablation study examining the influence of key parameters (such as number of capsules or routing depth) would strengthen the methodological section.

2. The evaluation currently compares RareCapsNet with several established rare-cell detection approaches such as FiRE, GiniClust, RaceID, and CellSIUS. While these comparisons are appropriate, the manuscript may benefit from additional comparisons with recent deep learning–based representation learning methods for single-cell data, such as autoencoder-based frameworks or graph neural network approaches.

3. The manuscript briefly discusses cross-dataset transferability of the learned capsule representations. This is an interesting property of the model, but the discussion could be expanded to provide more quantitative analysis and interpretation.

4. Since large-scale single-cell datasets often contain hundreds of thousands of cells, a short discussion about the computational cost of RareCapsNet would be useful. Providing approximate training time, memory requirements, or scalability characteristics would help readers assess how the method can be applied to very large datasets

5. The manuscript briefly discusses transferability of the model across different datasets. This is an interesting and potentially impactful property of RareCapsNet. The authors may consider expanding this section slightly to provide additional details about: how datasets were aligned or normalized before transfer, whether batch effects influence capsule activation patterns.

6. In some sections, terms such as cell types, cell classes, and type capsules are used interchangeably. Maintaining consistent terminology throughout the manuscript would improve clarity.

7. Some figure captions could be expanded to improve interpretability. In particular, figures showing capsule activations or coupling coefficients would benefit from more detailed descriptions explaining how readers should interpret the visual pattern.

8. Although the datasets used in the experiments are well known, it would be helpful to include a short table summarizing key properties of each dataset (number of cells, number of genes, number of cell types, proportion of rare populations).

9. The discussion section could be slightly expanded to emphasize broader implications of the framework and potential future extensions.

10. The manuscript mentions the availability of the implementation. Providing more details regarding dependencies, runtime environment, and example scripts would further facilitate adoption by the research community.

Reviewer #3: This manuscript proposed RareCapsNet, a method based on capsule networks for discovering rare cell populations with an emphasis on explainability. It is compared with GiniClust, FiRE, RaceID, and CellSIUS, using both simulated datasets and several real scRNA-seq datasets, such as Jurkat, CBMC, PBMC, and Mouse brain dataset. The topic is interesting and potentially important for rare cell type detecting in single-cell analysis. However, the manuscript requires substantial improvements in clarity, methodological description, and presentation before it can be considered further. My detailed comments are listed below:

Major concerns:

1. After carefully reading the manuscript, the overal framework of RareCapsNet is still difficult to understand. The procedures presented in Figure 1 is not sufficiently clear. The training objective (such as loss function) is not explicitly described in the text. I recommend that the authors could include a section describing the complete workflow in detail. In particular, the mathematical formulation of each layer (with its size) and the training procedure should be clearly defined.

2. Is RareCapsNet a supervised learning method? If the model relies on labeled data, its applicability for detecting rare cell types in new datasets may be limited. But the authors claimed that the method can identify "novel rare cell population" in the abstract. The authors should also clarify how to distinguish novel cell types with outliers.

3. I'm still confused with the concepts of "primary capsule", "type capsule". Does the number of primary capsules correspond to feature groups or feature types? These terms should be clearly defined with in the methodological workflow (a detailed workflow as comment 1).

4. The "dynamic routing procedure" is not clearly described? Does Equation (2) represent its formal definition? A clearer explanation would be helpful.

5. The manuscript claims explainability, but the definition is unclear. Is the explainability measured by the average correlation between primary capsules and cell-type capsules (as illustrated in Figure 1(D) and Eq.(1) in the Methods section)? Additionally, does the term 1/N(t) aim to compensate for differences in cell counts between cell types?

6. The figures require significant improvement.

(1) The text within the figures is too small and difficult to read. Important elements should be enlarged or highlighted.

(2) Some symbols are missing or unclear, e.g., the notation "N" in Figure 1(A) appears incomplete. Does "N" represent the number of cells with one feature to train? Please clarify the details. In Figure 1(B), it is unclear whether the input corresponds to a single cell or multiple cells. (As comment 1, a detailed workflow is necessary)

7. (1) In Data S2 and Data S4, the dropout rate is ~0.5 (approximately or exactly?) in the text. But it is reported as 0.4 in Table 1 and in SI. This inconsistency should be clarified.

(2) For Data S5, it is unclear whether "four samples" (used in the text) or "four classes" (used in the table) are simulated. The statement "lower than 10%" is inaccurate (for example 0.01 is also <10%).

8. The result shown in Figure 3(A) is confusing. Why does Leiden clustering fail to cluster in this case. (One cell belonging to Cluster-2 appears in Cluster 1, and three cells belonging to Cluster-1 are surrounded by Cluster-2.) More explanation is needed.

9. Page 7: "RareCapsNet successfully identified rare subpopulations". How to define "successful"? The criteria for defining successful identification should be clearly stated, especially for rare cell populations.

Page 7: "biologically meaningful gene sets". The authors should do the functional enrichment analysis to support this claim.

10. Page 7: (1) "These results confirm that RareCapsNet is suitable for cross-dataset applications without the need for full retraining."

The evidence (e.g. 85% or 91% accuracy for the whole dataset?) provided is not sufficient to support such a strong conclusion. The authors should present the results more objectively.

(2) "Yan19, Pollen20, Darmanis21, and CBMC22 (see supplementary text for the detailed description of the datasets)". I searched the word "Yan/Pollen..." in the SI, but could not find the corresponding descriptions in the SI. Please make a clear statement or citation.

(3) I supposed that CBMC used as the training dataset was labeled and obtained from the same tissue as the other three datasets. If the number of cell types differs across datasets, can the model detect new rare cell types that were not present during training?

Minor concerns:

The manuscript contains numerous typos and formatting issues. Adding line numbers would make it easier for reviewers to refer to specific parts of the text.

1. Mathematical symbols in figures are sometimes incorrectly formatted. E.g., subscripts or superscripts, such as "c_{ij}" in Figure 2 caption. The labels (A) and (B) are also missing in the figure 2. Please check the mathematical expressions in the manuscript thoroughly.

2. Page 5: "e.g. for setup S5 in supplementary figure, primary capsule 20 is activated for two types of cell samples."

Please specify the exact figure number in the SI.

3. Page 5: "We also compare the efficacy of RareCapsNet with other state-of-the-arts in simulation data".

Please list the other methods explicitly in the text.

4. Page5: "It can noticed that RareCapsNet outperforms other in terms of F1-score on most of the cases of simulation study."

Please report the results rigorously in the text (not by only one word "outperform"). The performance of comparision focuses mainly on F1-score. It would be useful to also report other metrics, such as ROC/AUC, accuracy, precision-recall curves, etc.

5. The dataset "293T cells" is mentioned but not clearly described. The maintext should provide basic dataset information, such as the number of cells and cell types of "Jurkat/293T cells". Now, it's difficult for the reader to get the key information of the dataset before reading the computational results.

6. The section organization is confusing. For example, under "Robust identification of rare cell type in poorly covered cells", separate subsections for Jurkat and CBMC experiments would improve clarity (missing subsection title?).

7. Page 7: retaining ¿85% accuracy

8. Page 9: All "c^s_{i, j}" are typos with additional ",".

9. Theorem 1 describes FDR control, which is a well-known statistical result. Please clarify whether the proof is original or based on existing literature.

10. The notation definitions in Page 9 and Page 13 are repeated.

11. Page 13: what does "Additional data" mean? It should be clarified.

12. The link to RareCapsNet on github is not accessible.

**********

**Have the authors made all data and (if applicable) computational code underlying the findings in their manuscript fully available?**

The PLOS Data policy requires authors to make all data and code underlying the findings described in their manuscript fully available without restriction, with rare exception (please refer to the Data Availability Statement in the manuscript PDF file). The data and code should be provided as part of the manuscript or its supporting information, or deposited to a public repository. For example, in addition to summary statistics, the data points behind means, medians and variance measures should be available. If there are restrictions on publicly sharing data or code —e.g. participant privacy or use of data from a third party—those must be specified.

Reviewer #1: None

Reviewer #2: None

Reviewer #3: **No:**

**********

PLOS authors have the option to publish the peer review history of their article (what does this mean?). If published, this will include your full peer review and any attached files.

If you choose “no”, your identity will remain anonymous but your review may still be made public.

**Do you want your identity to be public for this peer review?**  For information about this choice, including consent withdrawal, please see our Privacy Policy.

Reviewer #1: No

Reviewer #2: No

Reviewer #3: No

[NOTE: If reviewer comments were submitted as an attachment file, they will be attached to this email and accessible via the submission site. Please log into your account, locate the manuscript record, and check for the action link "View Attachments". If this link does not appear, there are no attachment files.]

**Figure resubmission:**

While revising your submission, we strongly recommend that you use PLOS’s NAAS tool (https://ngplosjournals.pagemajik.ai/artanalysis) to test your figure files. NAAS can convert your figure files to the TIFF file type and meet basic requirements (such as print size, resolution), or provide you with a report on issues that do not meet our requirements and that NAAS cannot fix.

After uploading your figures to PLOS’s NAAS tool - https://ngplosjournals.pagemajik.ai/artanalysis, NAAS will process the files provided and display the results in the "Uploaded Files" section of the page as the processing is complete. If the uploaded figures meet our requirements (or NAAS is able to fix the files to meet our requirements), the figure will be marked as "fixed" above. If NAAS is unable to fix the files, a red "failed" label will appear above. When NAAS has confirmed that the figure files meet our requirements, please download the file via the download option, and include these NAAS processed figure files when submitting your revised manuscript.

**Reproducibility:**

To enhance the reproducibility of your results, we recommend that authors of applicable studies deposit laboratory protocols in protocols.io, where a protocol can be assigned its own identifier (DOI) such that it can be cited independently in the future. Additionally, PLOS ONE offers an option to publish peer-reviewed clinical study protocols. Read more information on sharing protocols at https://plos.org/protocols?utm_medium=editorial-email&utm_source=authorletters&utm_campaign=protocols

Attachment Submitted filename: Comments to authors.docx

# Author comment: Author response to Decision Letter 1

**DOI:** 10.1371/journal.pcbi.1013962.r002

12 Jun 2026

Attachment Submitted filename: Point_by_Point_Response.pdf

# Peer review: Decision Letter 1

**DOI:** 10.1371/journal.pcbi.1013962.r003

**Authors:** Pedro Mendes, Xiaoqiang Sun

9 Jul 2026

Dear Dr. Ray,

We are pleased to inform you that your manuscript 'RareCapsNet: An explainable capsule networks enable robust discovery of rare cell populations from large-scale single-cell transcriptomics' has been provisionally accepted for publication in PLOS Computational Biology.

Before your manuscript can be formally accepted you will need to complete some formatting changes, which you will receive in a follow up email. A member of our team will be in touch with a set of requests.

Please note that your manuscript will not be scheduled for publication until you have made the required changes, so a swift response is appreciated.

IMPORTANT: The editorial review process is now complete. PLOS will only permit corrections to spelling, formatting or significant scientific errors from this point onwards. Requests for major changes, or any which affect the scientific understanding of your work, will cause delays to the publication date of your manuscript.

Should you, your institution's press office or the journal office choose to press release your paper, you will automatically be opted out of early publication. We ask that you notify us now if you or your institution is planning to press release the article. All press must be co-ordinated with PLOS.

Thank you again for supporting Open Access publishing; we are looking forward to publishing your work in PLOS Computational Biology.

Best regards,

Xiaoqiang Sun

Academic Editor

PLOS Computational Biology

Pedro Mendes

Section Editor

PLOS Computational Biology

***********************************************************

Reviewer's Responses to Questions

**Comments to the Authors:**

**Please note here if the review is uploaded as an attachment.**

Reviewer #1: I thank the author for providing answers to my questions and comments. The quality of manuscript has improved significantly.

Reviewer #2: The added details on the RareCapsNet architecture, ablation study, deep learning baseline comparisons, cross-dataset transfer analysis, computational cost, dataset summary, and implementation details have significantly improved the quality and reproducibility of the work.

Overall, the revisions are satisfactory. However, I recommend that the authors make minor revisions, mainly involving careful proofreading, checking figure/table numbering, ensuring supplementary materials are complete, and softening any remaining overly strong claims in their manuscript.

Reviewer #3: The authors have responsed to my major concerns, but I could not find responses to the minor concerns in the response letter. I would strongly encourage the authors to display all your changes (both major and minor ones) directly in the response letter. This is important because reviewers and editors may not have the opportunity to carefully compare every revision against the manuscript and re-examine the entire paper.

**********

**Have the authors made all data and (if applicable) computational code underlying the findings in their manuscript fully available?**

The PLOS Data policy requires authors to make all data and code underlying the findings described in their manuscript fully available without restriction, with rare exception (please refer to the Data Availability Statement in the manuscript PDF file). The data and code should be provided as part of the manuscript or its supporting information, or deposited to a public repository. For example, in addition to summary statistics, the data points behind means, medians and variance measures should be available. If there are restrictions on publicly sharing data or code —e.g. participant privacy or use of data from a third party—those must be specified.

Reviewer #1: Yes

Reviewer #2: None

Reviewer #3: None

**********

PLOS authors have the option to publish the peer review history of their article (what does this mean?). If published, this will include your full peer review and any attached files.

If you choose “no”, your identity will remain anonymous but your review may still be made public.

**Do you want your identity to be public for this peer review?**  For information about this choice, including consent withdrawal, please see our Privacy Policy.

Reviewer #1: No

Reviewer #2: No

Reviewer #3: No

# Editor report: Acceptance letter

**DOI:** 10.1371/journal.pcbi.1013962.r004

**Authors:** Pedro Mendes, Xiaoqiang Sun

PCOMPBIOL-D-26-00254R1

RareCapsNet: An explainable capsule network enables robust discovery of rare cell populations from large-scale single-cell transcriptomics

Dear Dr Ray,

I am pleased to inform you that your manuscript has been formally accepted for publication in PLOS Computational Biology. Your manuscript is now with our production department and you will be notified of the publication date in due course.

The corresponding author will soon be receiving a typeset proof for review, to ensure errors have not been introduced during production. Please review the PDF proof of your manuscript carefully, as this is the last chance to correct any errors. Please note that major changes, or those which affect the scientific understanding of the work, will likely cause delays to the publication date of your manuscript.

Soon after your final files are uploaded, unless you have opted out, the early version of your manuscript will be published online. The date of the early version will be your article's publication date. The final article will be published to the same URL, and all versions of the paper will be accessible to readers.

For Research, Software, and Methods articles, you will receive an invoice from PLOS for your publication fee after your manuscript has reached the completed accept phase. If you receive an email requesting payment before acceptance or for any other service, this may be a phishing scheme. Learn how to identify phishing emails and protect your accounts at https://explore.plos.org/phishing.

Thank you again for supporting PLOS Computational Biology and open-access publishing. We are looking forward to publishing your work!

With kind regards,

Sharmila Kamatchi

PLOS Computational Biology | Carlyle House, Carlyle Road, Cambridge CB4 3DN | United Kingdom ploscompbiol@plos.org | Phone +44 (0) 1223-442824 | ploscompbiol.org | @PLOSCompBiol
